# Acclimation temperature affects thermal reaction norms for energy reserves in *Drosophila*

**DOI:** 10.1038/s41598-020-78726-z

**Published:** 2020-12-10

**Authors:** Peter Klepsatel, Thirnahalli Nagaraj Girish, Martina Gáliková

**Affiliations:** 1 Institute of Zoology, Slovak Academy of Sciences, Dúbravská cesta 9, 845 06 Bratislava, Slovakia; 2 Centre of Biosciences, Slovak Academy of Sciences, Dúbravská cesta 9, 840 05 Bratislava, Slovakia; 3 Department of Biosciences, Sri Sathya Sai Institute of Higher Learning, 515134 Prasanthi Nilayam, India

**Keywords:** Ecophysiology, Fat metabolism

## Abstract

Organisms have evolved various physiological mechanisms to cope with unfavourable environmental conditions. The ability to tolerate non-optimal thermal conditions can be substantially improved by acclimation. In this study, we examined how an early-life acclimation to different temperatures (19 °C, 25 °C and 29 °C) influences thermal reaction norms for energy stores in *Drosophila* adults. Our results show that acclimation temperature has a significant effect on the amount of stored fat and glycogen (and their relative changes) and the optimal temperature for their accumulation. Individuals acclimated to 19 °C had, on average, more energy reserves than flies that were initially maintained at 25 °C or 29 °C. In addition, acclimation caused a shift in optimal temperature for energy stores towards acclimation temperature. We also detected significant population differences in this response. The effect of acclimation on the optimal temperature for energy stores was more pronounced in flies from the temperate climate zone (Slovakia) than in individuals from the tropical zone (India). Overall, we found that the acclimation effect was stronger after acclimation to low (19 °C) than to high (29 °C) temperature. The observed sensitivity of thermal reaction norms for energy reserves to acclimation temperature can have important consequences for surviving periods of food scarcity, especially at suboptimal temperatures.

## Introduction

Since all biological processes are influenced by temperature, any change in the thermal environment has a strong effect on the animal’s physiology^1–3^. Acclimation is a physiological response to environmental changes that reduces stress and/or improves organismal performance^4,5^. Thermal acclimation may encompass multiple, diverse responses, such as changes in membrane lipid composition (e.g. Ref.^6,7^), enzyme activities (e.g.^8,9^), expression of heat-shock proteins (e.g.^10,11^), or behavioural changes (e.g.^12^). Acclimation is usually the result of long-term exposure (days or weeks), as opposed to hardening, which is the physiological reaction to a brief exposure to a stressful environment^13,14^. According to Collier et al*.*^15^, acclimation responses occur in two phases. During the first, acute phase, various metabolic and physiological responses (including heat shock response) take place at the cellular and systemic level; the second, long-term phase involves substantial changes in transcriptome, proteome and metabolome (e.g.^16–18^). However, not all changes elicited by temperature, or any other environmental stimuli, are necessarily adaptive; some alterations are just a result of stressful conditions^19^. For example, prolonged exposure to low or high temperatures can cause cellular damage and apoptosis (e.g.^20^).

The relationship between temperature and a physiological process is typically described by a thermal performance curve, which is usually unimodal and often asymmetrical^21–23^. Thermal performance initially rises with temperature, reaches a maximum at the optimal temperature, and gradually decreases with a further temperature increase^21–23^. Both position and shape of the thermal performance curve can be altered by acclimation^23,24^. Theoretically, the beneficial effect of thermal acclimation can lead to an adaptive shift in the thermal performance (i.e. the optimal temperature) towards the acclimation temperature^25^. Although the adaptive character of acclimation is well-documented in the case of thermal tolerance (e.g.^7,17,26–31^), the evidence for beneficial effects of thermal acclimation on other fitness-related traits is often contradictory (e.g.^4,32–37^). According to Ayrinhac et al*.*^27^, the fact that acclimation might have opposite effects on different traits or physiological processes only demonstrates “the complexity and diversity of plastic responses”.

The animal’s ability to survive periods of food shortage depends strongly on the amount of stored fat and glycogen^38^. Under optimal conditions, the positive energy balance allows accumulation of energy stores, whereas suboptimal or stressful conditions lead to their reduction^39–41^. Previous studies in the fruit fly *Drosophila melanogaster* have shown that the fat and glycogen content is significantly influenced by temperature^40,41^. The relationship between temperature and the amount of stored lipids and glycogen can be described by a quadratic function with a maximum value reached at approximately 21°C^41^. At intermediate temperatures (15–27 °C), flies can build up energy stores, whereas exposure to lower or higher temperatures decreases the amount of stored fat and glycogen^41^. In the present work, we have tested whether early-life acclimation affects thermal reaction norms for energy reserves in *D. melanogaster*. We have used outbred flies from two populations originating from distinct climate zones: tropical (India) and temperate (Slovakia). Experimental individuals were acclimated during early life to three different temperatures (19, 25 and 29 °C), subsequently exposed as adults to eleven temperatures in the range of 11 °C to 31 °C, and examined for the amount of stored fat and glycogen. We have hypothesised that acclimation might significantly affect thermal reaction norms for energy stores. Based on the assumptions of the beneficial acclimation hypothesis^4,25,42^, acclimation might shift these reaction norms in accordance with the temperature of acclimation, i.e. flies acclimated to a lower temperature might have a lower optimal temperature for energy stores than individuals acclimated to a higher temperature (and vice versa). Finally, we have also examined whether temperature-induced changes in fat reserves are associated with underlying changes in the lipid droplets—fat storage organelles^43,44^.

## Results

### Acclimation temperature affects thermal reaction norms for fat reserves

To examine the effect of acclimation temperature on thermal reaction norms for energy reserves and to account for potential intraspecific differences, we used individuals from outbred, wild-caught populations of *D. melanogaster* from the temperate (Slovakia) and the tropical (India) climate zone. Since our previous study^41^ has shown that the relationship between temperature and the amount of stored fat and glycogen is best described by a quadratic function, we used multiple nonlinear (quadratic) regression to analyse the effects of population origin, the acclimation and test temperatures, and their interactions on the amount of stored fat. All the factors, including their interactions (except two three-way interactions), had a significant effect on both the absolute fat content and the relative changes in the fat content (ratio between the fat content measured after and before exposure to a given temperature) (Supplementary Tables S1, S2). Similar results were also obtained for the fat content normalised to protein content (Supplementary Tables S3, S4).

**Figure 1:**
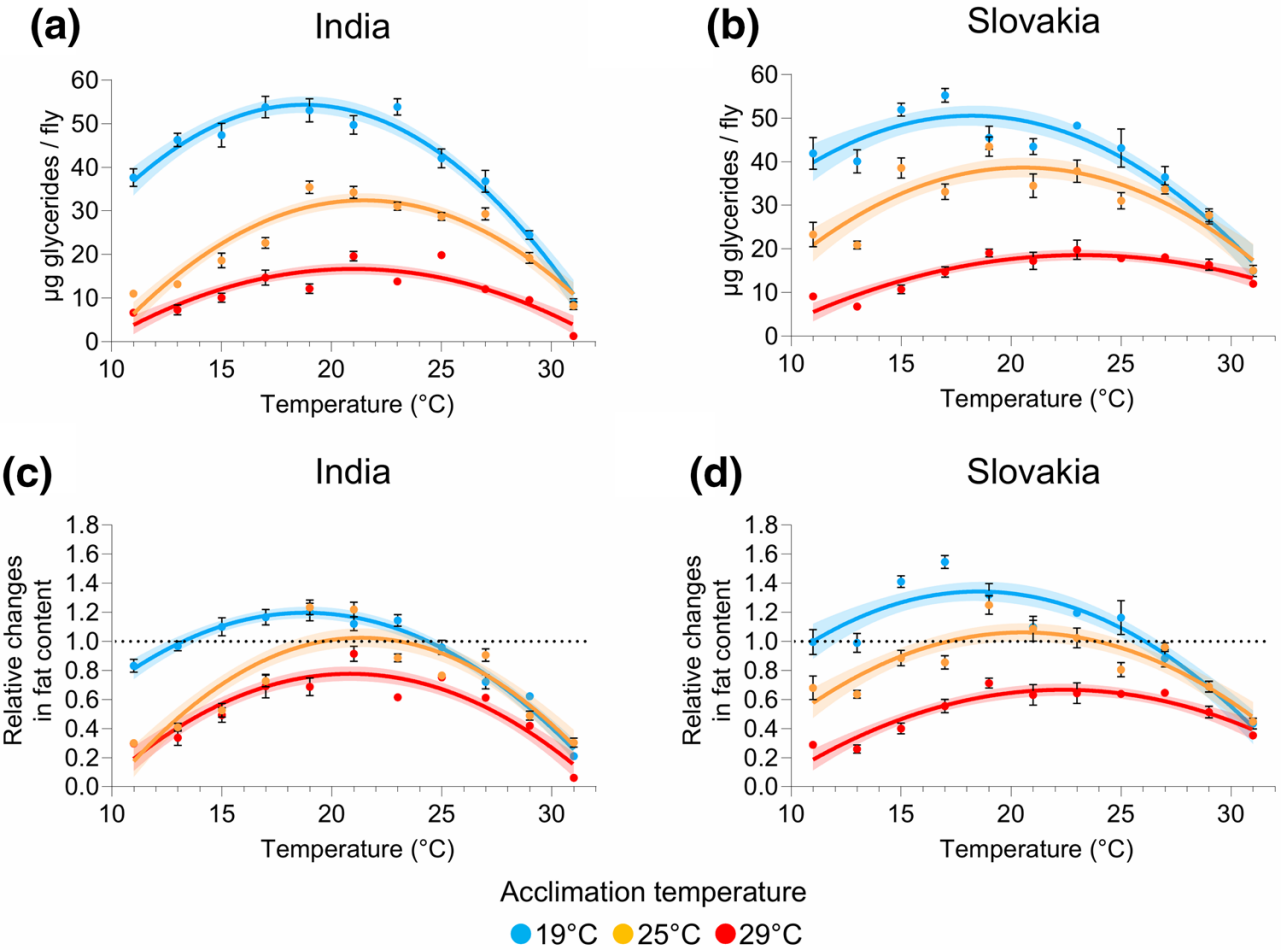
Acclimation temperature affects thermal reaction norms for fat reserves. (**a**, **b**) Absolute fat content (µg glycerides per fly) in flies from India (**a**) and Slovakia (**b**). (**c**, **d**) Relative changes in the absolute fat content (calculated as a ratio between the final values and the mean of initial values) in flies from India (**c**) and Slovakia (**d**). Data points are mean values ± s.e.m.. Lines represent a quadratic fit to data with the 95% confidence band. For statistical analyses, see Supplementary Tables S1, S2, S5.

In order to find shared patterns and potential differences between the populations, we analysed the effect of acclimation and test temperatures on the fat content for each population separately (Supplementary Table S5). Overall, individuals from both populations that were reared at 19 °C before the exposure to different test temperatures had, on average, a higher amount of stored fat than flies that were initially maintained at higher temperatures (Fig. 1a,b; Supplementary Fig. S1). Consistently, the cold-acclimated flies had significantly higher estimated maximum value of fat content (Supplementary Fig. S2). Importantly, individuals from both populations that were acclimated to 19 °C increased their fat content when exposed to temperatures between 15 °C and 23/25 °C, whereas flies that were acclimated to 25 °C were able to accumulate fat only when exposed to a temperature around 19–21 °C (Figs. 1c,d; S3). In contrast, the amount of stored fat decreased at all experimental temperatures in individuals acclimated to 29 °C (Fig. 1c,d; Supplementary Fig. S3).

**Figure 2:**
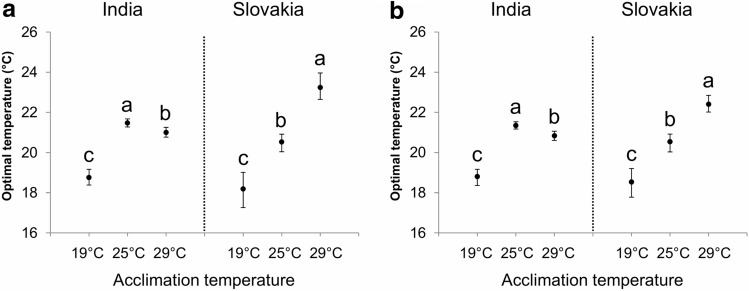
The optimal temperatures for fat reserves estimated based on (**a**) the absolute fat content (µg glycerides per fly), and (**b**) the relative changes in the absolute fat content. Error bars represent 95% confidence intervals. Values with different letters are significantly different from each other (α = 0.05).

Based on the fitted curves for the absolute fat content and the relative changes in the fat content, we also calculated the optimal temperature (i.e. the temperature at which the given trait reaches its maximum value), 75% performance breadth (i.e. the thermal range over which the amount of stored fat was at least 75% of the maximum value), and individual parameters of each reaction norm (quadratic coefficient, slope, and intercept). In general, there were only small differences among the estimated optimal temperatures for the absolute fat content (Fig. 2a), the relative changes in the fat content (Fig. 2b), the absolute fat content normalised to protein (Supplementary Fig. S4a), or the relative changes in the fat content normalised to protein (Supplementary Fig. S4b). In the Indian population, flies that were initially kept at 25 °C had a higher optimal temperature for the amount of stored fat (the calculated optimal temperature for the absolute fat content/the relative changes in the fat content is 21.5 °C/21.3 °C) than flies that were acclimated to either 19 °C (18.8 °C/18.8 °C) or 29 °C (21.0 °C/20.8 °C) (Fig. 2). In contrast, flies from the Slovak population that were initially maintained at 29 °C had a higher optimal temperature (23.2 °C/22.4 °C) than flies acclimated to 25 °C (20.5 °C/20.5 °C); individuals acclimated to 19 °C had the lowest optimal temperature for the amount of stored fat (18.2 °C/18.5 °C) (Fig. 2). These results are consistent also with those calculated based on the fat content normalised to protein content (Supplementary Fig. S4). In the Indian population, we also found significant differences in the performance breadth; flies acclimated to low temperature had a broader performance breadth than those acclimated to higher temperatures (Supplementary Fig. S5). Such trend was relatively weak or absent in flies from the Slovak population. Consistent with the above-mentioned findings, we detected significant differences also in the parameters of individual thermal reaction norms (Supplementary Fig. S6, S7). Overall, there seemed to be a weak trend towards a less concave (i.e. less steep) thermal reaction norm at higher acclimation temperature (as indicated by the significantly lower absolute value of the quadratic coefficient—*a*) (Supplementary Figs. S6a,d, S7a,d).

### Acclimation temperature has a significant impact on thermal reaction norms for glycogen stores

Using the same approach as for the fat reserves, we next examined the glycogen stores (Supplementary Tables S6–S10). The population, the acclimation and test temperatures, and three of their interactions (population × acclimation temperature; acclimation temperature × test temperature; acclimation temperature × (test temperature)^2^) significantly affected the absolute glycogen content and also the relative changes in the glycogen content (Supplementary Tables S6, S7). In the case of glycogen content normalised to the amount of proteins, we obtained similar outcome (Supplementary Tables S8, S9); however, two of the interactions (acclimation temperature × test temperature; acclimation temperature × (test temperature)^2^) were not statistically significant when we analysed the relative changes in the glycogen stores normalised to protein content (Supplementary Table S9).

**Figure 3:**
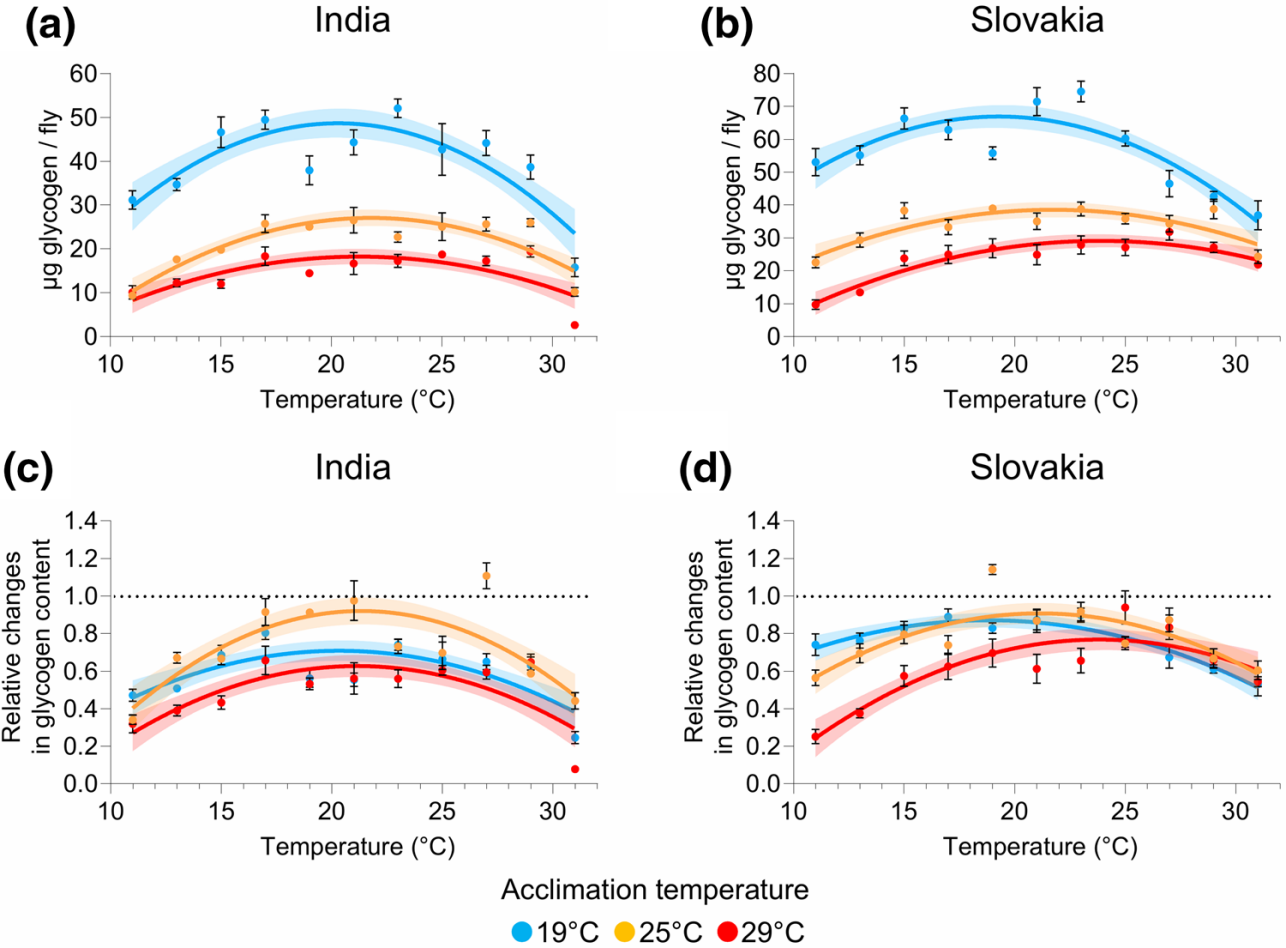
Acclimation temperature has a significant effect on thermal reaction norms for glycogen stores. (**a**, **b**) Glycogen content (µg glycogen per fly) in flies from India (**a**) and Slovakia (**b**). (**c**, **d**) Relative changes in the glycogen content (calculated as a ratio between the final values and the mean of initial values) in flies from India (**c**) and Slovakia (**d**). Data points are mean values ± s.e.m.. Lines represent a quadratic fit to data with the 95% confidence band. For statistical analyses, see Supplementary Tables S6, S7, S10.

**Figure 4:**
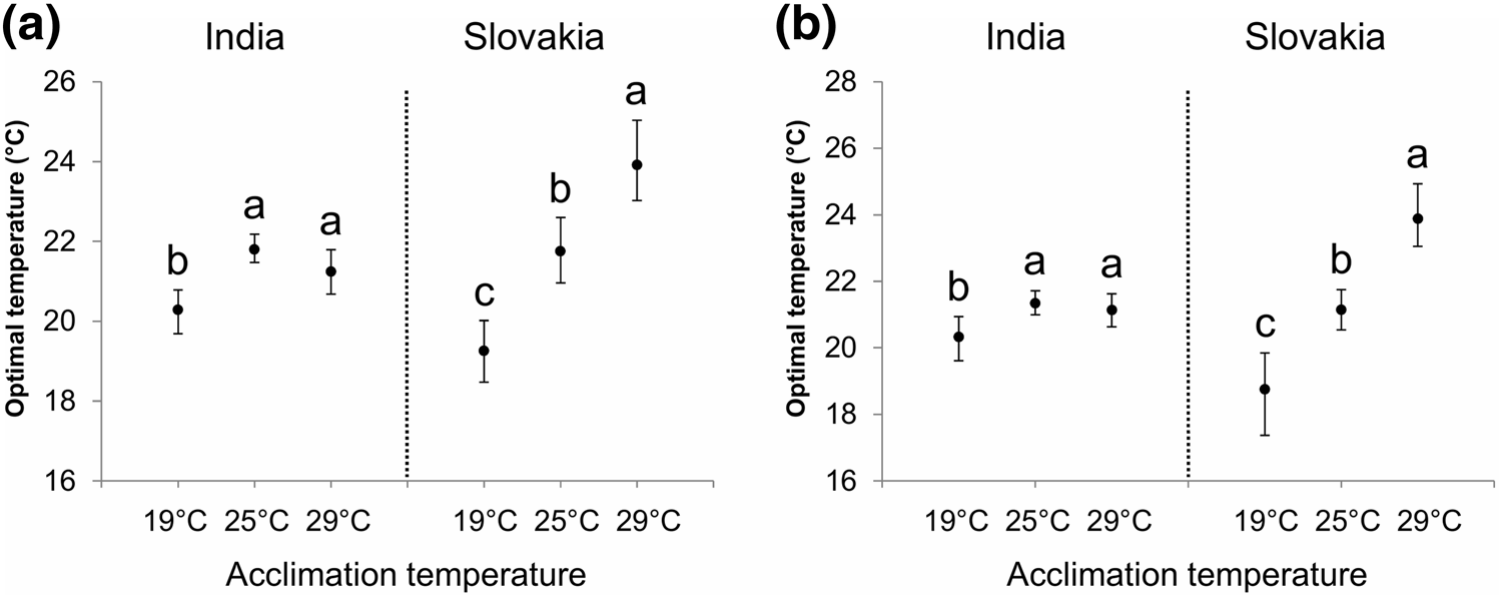
The optimal temperatures for glycogen stores estimated based on (**a**) the absolute glycogen content (µg glycogen per fly), and (**b**) the relative changes in the absolute glycogen content. Error bars represent 95% confidence intervals. Values with different letters are significantly different from each other (α = 0.05).

Similar to fat stores, the amount of glycogen (as well as the glycogen content normalised to protein content) (Fig. 3a, b; Supplementary Fig. S8), and its estimated maximum values (Supplementary Fig. S9) were inversely related to the acclimation temperature. In contrast, the glycogen content tended to decrease at all temperatures (Fig. 3c,d; Supplementary Fig. S10). Moreover, in comparison to fat stores, the acclimation temperature seemed to have a weaker (but still significant) effect on the relative changes in glycogen content (Fig. 3c,d; Supplementary Fig. S10). Nevertheless, at temperatures below 25 °C, the individuals that were initially kept at 29 °C seemed to deplete their glycogen reserves slightly more than the flies acclimated to 19 °C and 25 °C (Fig. 3c,d; Supplementary Fig. S10). The estimated optimal temperatures for the glycogen content and the relative changes in the glycogen content were very similar to those obtained based on the changes in fat reserves. Indian flies acclimated to 25 °C had comparable optimal temperatures for glycogen reserves (the calculated optimal temperature for the absolute glycogen content/the relative changes in the glycogen content: 21.8 °C/21.3 °C) as flies acclimated to 29 °C (21.2 °C/21.1 °C), whereas the estimated optimal temperatures for individuals acclimated to 19 °C was slightly lower (20.3 °C/20.3 °C) (Fig. 4). The effect of the acclimation temperature on the optimal temperature for glycogen reserves was again more pronounced in the Slovak population; there was a clear positive relation between the acclimation temperature and the optimal temperature (acclimation at 19 °C: optimal temperature = 19.3 °C/18.7 °C; acclimation at 25 °C: optimal temperature = 21.8 °C/21.1 °C; acclimation at 29 °C: optimal temperature = 23.9 °C/23.9 °C) (Fig. 4). Analogous results were obtained for the glycogen content normalised to protein content (Supplementary Fig. S11). Unlike the fat reserves, the acclimation temperature did not have substantial effect on the performance breadth (Supplementary Fig. S12). Finally, we did not find any clear trend in the estimated parameters of thermal reaction norms for glycogen stores in response to acclimation temperature (Supplementary Figs. S13, S14). Moreover, when the relative changes in glycogen stores were compared, the differences among the parameters were small or absent (Supplementary Figs. S13d–f, S14d–f).

### Temperature affects lipid droplet size and the relative cell area occupied by lipid droplets

**Figure 5:**
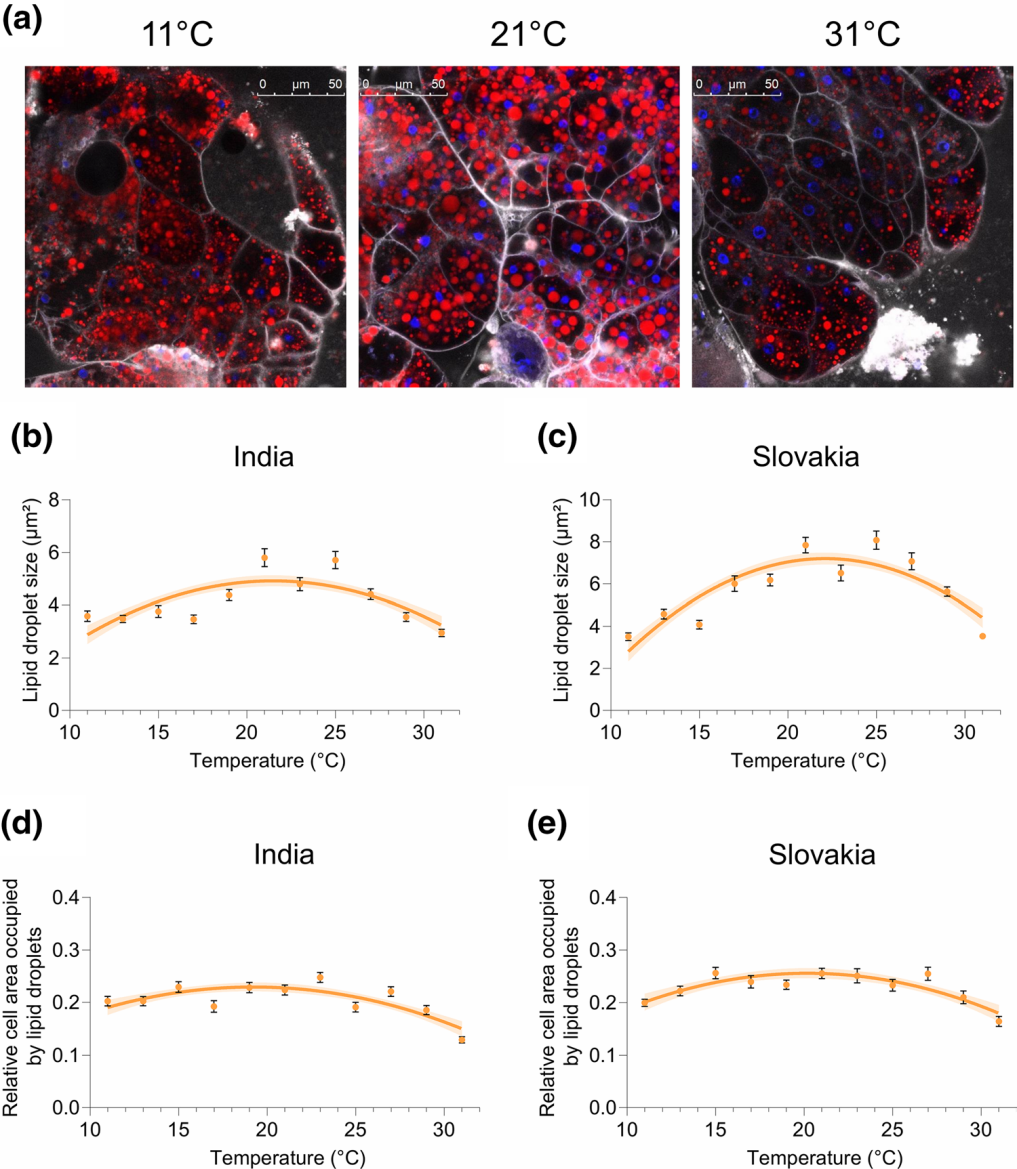
Temperature affects lipid droplet size and the relative cell area occupied by lipid droplets. (**a**) Confocal images of the fat body of *Drosophila* exposed to different temperatures; lipid droplets in red (Bodipy 493/503), cell membranes in white/grey (CellMask Deep Red), and DNA in blue (Hoechst 33342). (**b**, **c**) The relationship between temperature and lipid droplet size in flies from India (**b**) and Slovakia (**c**). (**d**, **e**) The effect of temperature on the relative cell area occupied by lipid droplets (a ratio between the area covered by lipid droplets and the total area of a given cell measured in a single optical section) in flies from India (**d**) and Slovakia (**e**). Data points are mean values ± s.e.m.. Lines represent a quadratic fit to data with the 95% confidence band. For statistical analyses, see Supplementary Tables S11, S12.

In insects, the central storage deposit of lipids is the fat body^45^. Fat is stored in the fat body adipocytes in specialised cellular organelles—lipid droplets (e.g.^43^). Lipid droplets are sensitive to changes in metabolism and nutritional status^44^. We, therefore, investigated to what extent the ambient temperature affects their size and relative volume (as a proxy we used the relative cell area occupied by lipid droplets measured in a cross-section) in the fat body adipocytes. We found that the relationship between temperature and the lipid droplet size, and the relative cell area occupied by lipid droplets can be described by a unimodal (quadratic) function (Supplementary Table S11). Temperatures below 15 °C or above 29 °C decreased both the size (Fig. 5a–c) and the relative cell area occupied by lipid droplets, i.e. adipocytes contained smaller lipid droplets at these presumably non-optimal temperatures, and lipid droplets tended to occupy (especially at 31 °C) a smaller relative volume in a cell (Fig. 5a,d,e). Interestingly, we also detected significant intraspecific differences in the thermal reaction norms for lipid droplet size (indicated by significant interactions between ‘population’ and ‘test temperature’, and ‘population’ and ‘(test temperature)^2^’); in the Slovak population, the relationship between temperature and the lipid droplet size was described by a quadratic function that was more concave (i.e. more pronounced differences between the lipid droplet size at intermediate vs low/high temperatures) than in the Indian population (Fig. 5b,c; Supplementary Table S12). In contrast, we did not detect any significant intraspecific differences in the thermal reaction norms for the relative cell area occupied by lipid droplets (Fig. 5d,e; Supplementary Table S12). Finally, we observed that the changes in lipid droplet size and the relative cell area occupied by lipid droplets are consistent, to some extent, with the temperature-induced changes in the amount of stored fat (as indicated by significant positive correlations between these variables) (Supplementary Table S13).

## Discussion

In this study, we examined how acclimation temperature affects thermal reaction norms for energy reserves in *Drosophila* adults. In general, the accumulation of energy reserves can occur only when energy intake outweighs energy expenditure, and in comparison to other energy-requiring processes, such as growth or reproduction, the accumulation of energy stores is of relatively lower priority^39^. Thus, increased deposition of fat and glycogen might indicate optimal environmental conditions^39^. Conversely, their significant reduction may be a marker of stressful conditions^39,40,46,47^. The amount of stored fat and glycogen might, therefore, represent an important indicator of physiological state^39^. Our results show that the acclimation temperature affects the absolute fat and glycogen content (and their relative changes) and the optimal temperature for their accumulation. Overall, the shape of the thermal reaction norms for fat stores tended to be more sensitive to acclimation temperature than thermal reaction norms for glycogen reserves. Individuals from both examined populations that were exposed to low temperatures early in life had, on average, higher fat and glycogen content than flies acclimated to 25 °C or 29 °C. In addition, the estimated optimal temperature for both fat and glycogen was lower in flies that were acclimated to 19 °C than in flies that were acclimated to higher temperatures (25 °C or 29 °C). Such a shift in the optimal temperature towards acclimation temperature might be adaptive and in accordance with the beneficial acclimation hypothesis^4,25^. In contrast, the performance breadth was affected by acclimation only in the case of fat reserves in the Indian flies.

Although theory suggests that acclimation may alter different parameters of the thermal performance curve, optimal temperature and maximum performance are usually less sensitive to acclimation than performance breadth^28,48^. For example, Deere and Chown^25^ found only minor variation in optimal temperature for locomotor performance, and no consistent changes in maximum performance and performance breadth in response to acclimation in five mite species. Other studies in different arthropods did not find any association between acclimation and optimal temperature^28,49–51^. On the other hand, shifts in optimal temperature or changes in maximum performance and/or performance breadths in response to acclimation have been repeatedly documented in ectothermic vertebrates (e.g.^52–54^). The substantial interspecific variation in acclimation responses may result from differences in selection pressures, thermoregulatory abilities, intrinsic constraints, and environmental predictability (reviewed in^55^). In addition, acclimation can have a different effect on different physiological functions^56^. Since complex traits depend on numerous physiological processes that might be affected by acclimation in different ways, the overall effect of acclimation on these traits might be neutral or even negative^56^. Altogether, in comparison to other studies on various traits in arthropods^25,28,49–51^, our results show that thermal reaction norms for energy reserves in *Drosophila* are relatively sensitive to acclimation.

Interestingly, we also found significant intraspecific differences in the acclimation response. The effect of acclimation on the optimal temperature for energy stores was more prominent in the Slovak population. In the Indian population, individuals acclimated to 29 °C had lower or comparable optimal temperatures for fat and glycogen in comparison to flies acclimated to 25 °C; whereas the optimal temperature for energy reserves for flies from Slovakia that were acclimated to 29 °C was higher than for individuals initially maintained at 25 °C. Another difference was that the estimated thermal performance breadth for fat reserves in flies from the Indian population was significantly narrower at higher acclimation temperatures (25 °C and 29 °C) than at 19 °C; in flies from the Slovak population, such trend was relatively weak or absent. These findings might indicate that flies from Slovakia have greater acclimation ability than the Indian flies. According to Gabriel and Lynch^57^, organisms that experience higher levels of environmental variance between generations may have a greater capacity to acclimate in comparison to organisms from more stable environments. Consequently, organisms from the temperate climate zone, which experience seasonal temperature fluctuations, should exhibit higher thermal plasticity than organisms from the tropical zone (e.g.^58–60^). Moreover, this effect should be more pronounced in species that complete several generations per year (such as *Drosophila*) where offspring may encounter different thermal conditions than their parents did^57,61^. Experimental studies, however, provide mixed support for this idea (e.g.^58,60–63^). For example, temperate mayflies (Ephemeroptera) tend to have greater acclimation ability than tropical mayflies^60^. In contrast, Hoffmann and Watson^62^ did not detect any significant differences in the acclimation response to heat and cold in tropical and temperate populations of *D. melanogaster* and *D. simulans*. Similarly, Cooper et al*.*^58^ found no evidence for divergence in thermal acclimation capacity of fecundity in *D. melanogaster* from a highly versus less seasonal environment. Overall, it seems that the majority of studies in *Drosophila* do not provide strong evidence for intraspecific variation in acclimation capacities^58,61–63^. Because we examined flies from only two populations, further study with a larger number of populations is needed before making broader generalizations about differences in the acclimation response of energy stores in individuals from different climate zones.

In accordance with the previous studies^40,41^, we found that exposure to non-optimal temperatures can cause a substantial depletion of energy reserves, which might have a significant negative impact on survival in times of food scarcity^40^. Despite the observed shift in the optimal temperature, thermal acclimation did not seem to have an effect on the rate of depletion of energy stores at higher temperatures. In contrast, acclimation to 19 °C appeared to substantially diminish the reduction of fat reserves at low temperatures (< 15 °C). This finding might correspond with previous studies indicating that low-temperature tolerance is more responsive to acclimation than tolerance to high temperature (e.g.^28,64,65^). Moreover, cold-acclimated flies were able to accumulate additional fat at intermediate temperatures, whereas warm-acclimated (29 °C) individuals depleted their fat reserves even at presumably optimal thermal conditions. This observation supports the notion that whereas acclimation to non-stressful environments can have beneficial effects on fitness, exposure to stressful conditions (high temperature in this case) during early life might have a long-term negative impact on adult performance^66^. Although the underlying cause of this phenomenon is unclear, it may involve the increased rate of cell death of the fat body adipocytes^40^ or their reduced capacity to store lipids.

Fat reserves are stored in specialised cellular organelles, lipid droplets^43^ that occur predominantly in cells (adipocytes) of the fat body, which is the major organ of fat storage in insects^45^. Number and size of lipid droplets increase or decrease in response to nutrient availability^44^, and therefore, these characteristics are sometimes used as indicators of imbalance between energy intake and energy expenditure. Since the effect of temperature on the abundance and size of the lipid droplets has not been analysed yet, we tested whether and how these organelles respond to ambient temperature. In agreement with the results on the fat content, we found a unimodal relationship between temperature and both the lipid droplet size and the relative cell area occupied by lipid droplets. We found that exposure to either lower (˂ 15 °C) or higher (˃ 29 °C) temperatures decreases lipid droplet size, as well as the relative area occupied by lipid droplets in the fat body cells. Although our study did not test how thermal acclimation affects lipid droplets, it is evident that the size and abundance of these organelles respond to ambient temperature. Since the cellular mechanisms that regulate lipid droplet size and numbers are still poorly understood^43,44^, it is not clear whether the fat body cells store less lipids at non-optimal temperatures due to damage or inhibition of formation of lipid droplets, or simply due to a shifted balance between energy intake and expenditure. Moreover, it seems that these changes might not be solely responsible for the observed changes in the total fat content; there are very likely also other processes involved, such as changes in the number of fat body adipocytes^40^. However, the fact that temperature affects characteristics of lipid droplets might suggest that the temperature-driven changes in fat reserves are, at least partially, caused by the altered balance between energy input and output. As has been discussed previously^41^, there are several mechanisms that could potentially explain the temperature-driven changes in energy stores. For example, it has been observed that stressful temperatures induce apoptosis in *Drosophila* adipocytes, which can cause a decline in body fat^40^. Non-optimal conditions may also impose on organism additional energetic costs, for instance, due to increased proteolysis and protein synthesis, metabolic perturbations, damage repair etc.^17,31,39,67^. Other potential causes that can lead to increased utilisation of energy reserves include lower efficiency of energy acquisition^68,69^ and reduced assimilation efficiency (the relative amount of energy obtained per unit of consumed food)^70^. Previous studies indicate that the temperature-induced changes in energy stores in *Drosophila* might result from a combination of altered assimilation efficiency and stress-induced apoptosis^40,41^.

In summary, we found that early-life acclimation significantly affects the absolute amount of stored fat and glycogen. Moreover, we revealed that the optimal temperature for energy reserves positively correlates with the acclimation temperature. Our findings suggest that whereas cold-acclimation has a beneficial effect on overall energy balance at low temperatures, acclimation to high temperature (29 °C) has rather deleterious effects on physiological processes related to energy storage. Overall, our results demonstrate that thermal reaction norms for *Drosophila* energy reserves are sensitive to acclimation temperature, which can have a direct impact on survival and fitness, especially under nutritionally poor conditions.

## Materials and methods

### Fly populations and maintenance

We used two wild-caught populations of *D. melanogaster* from the tropical (collected in Mysore, Karnataka, India; July 2017), and the temperate (collected in Bratislava, Slovakia; October 2017) climate zone. Both laboratory populations were initiated from at least 300 freshly collected flies and were kept in the laboratory as outbred populations (at population sizes of approx. 1500–2000 adults) at 25 °C (12 h:12 h light–dark cycle, 60% relative humidity) for 21 (population from India)/ 18 (population from Slovakia) months prior to the experiments (for further details see^32^). All flies (including experimental individuals) were maintained on a standard *Drosophila* medium (6 g agar, 50 g yeast, 50 g sucrose, 70 g maize flour, 5.12 ml propionic acid and 1.3 g methylparaben per 1 L of medium).

### Thermal treatments

To obtain experimental individuals for testing the effect of acclimation temperature on the amount of stored fat and glycogen, parental flies (approx. one week old; approx. 100 individuals) were allowed to lay eggs into vials (68 ml) containing standard medium during a three-hour period. Vials with intermediate egg density (approx. 150 eggs per 68 ml vial) were placed either in an incubator at 19 °C, 25 °C, or 29 °C. Males collected within 12 h after emergence were placed on the standard medium (approx. 30 males per 68 ml vial; three vials per acclimation temperature, test temperature, and population) and kept at the same temperature as under development for four days. This additional period was necessary to ensure complete removal of the larval fat body, which is still present in newly eclosed flies but depleted and replaced within few days after eclosion^71,72^. The four-day old experimental individuals were then exposed to one of eleven temperatures (11 °C, 13 °C, 15 °C, 17 °C, 19 °C, 21 °C, 23 °C, 25 °C, 27 °C 29 °C, 31 °C) at a 12 h:12 h light–dark cycle, and 60% relative humidity. Flies were maintained at their given experimental temperatures for eight days with daily food exchange. Samples for the lipid and glycogen quantification (five males per sample; six samples (two samples from each vial) per acclimation temperature, test temperature, and population) were collected at the beginning and at the end of this eight-day period^41^.

### Fat and glycogen quantification

To determine the amount of fat (glycerides), glycogen and proteins, we used 4–6 samples (5 males per sample) per acclimation temperature, test temperature, and population. Samples were homogenised in 600 μL of 0.05% Tween-20 using TissueLyser II (Qiagen) at 30 s^−1^ for 1 min., heat-inactivated at 70 °C for 5 min and centrifuged at 3000g for 3 min^73^. Fat content was determined by a coupled colorimetric assay using the Triglycerides (liquid) assay (Randox, TR1697) as described in Gáliková et al.^74^. Fat content is expressed either as absolute fat content (μg glycerides per fly) or as fat content normalised to protein content (μg glycerides per mg protein)^75^. The quantification of proteins was performed using the Pierce Coomassie (Bradford) Protein Assay Kit (Thermo Scientific, 23200) according to the manufacturer's protocol. The amount of glycogen was measured by the GO kit (Sigma, GAGO)^75^ as described in Gáliková et al.^74^. Relative changes in fat or glycogen content were calculated as a ratio between the final value (measured at the end of the eight-day period of exposure to a given temperature) and the average of initial values (measured right before the exposure to a given temperature). Values > 1 signify accumulation of fat or glycogen, whereas values < 1 indicate depletion of energy reserves.

### Lipid droplet analysis

Lipid droplets were analysed in males that were initially kept at 25 °C and then exposed to eleven different temperatures for eight days as described above. To stain lipid droplets in the fat body, 10–12 individuals were dissected in 1xPBS (phosphate-buffered saline), and their carcass was subsequently embedded in 1xPBS containing Bodipy 493/503 (1:100; Invitrogen, D3922) for visualising lipid droplets, Hoechst 33342 (1:100; Invitrogen, H3570) for nuclei staining, and CellMask Deep Red (1:100; Invitrogen, C10046) for plasma membrane staining. Samples were viewed on a confocal microscope Leica TCS SPE (Leica Microsystems, Germany). Lipid droplets were examined as described in Gáliková et al*.*^76^. In brief, confocal images were analysed using ImageJ v1.52p. In order to smooth the edges of lipid droplets, a Gaussian blur function (2 pixel radius) was applied to a single optical section. Next, a binary image was created by automatic thresholding; clustered lipid droplets were separated by applying watershed segmentation. Lipid droplet size was measured by a particle analyser (size (µm^2^): 0.0 → ∞; circularity: 0.01–1.0). For each test temperature and population, lipid droplets from 40–60 cells (originated from several different individuals, approx. 4–12) were analysed. The relative cell area occupied by lipid droplets was calculated as a ratio between the cell area covered by lipid droplets and the area of a given cell in a single optical section.

### Statistical analyses

The previous study^41^ has shown that the relationship between temperature and energy reserves is best described by a quadratic function. Thus, to examine the effect of thermal acclimation and population origin on thermal reaction norms for the amount of fat and glycogen, or for the relative changes in their amounts, we used multiple nonlinear (polynomial—quadratic) regression analyses with two categorical factors (‘acclimation temperature’, ‘population’), two continuous factors (‘test temperature’, ‘(test temperature)^2^’), and seven interactions (see Tables S1–S4, S6–S9). In addition, we also performed similar analyses for each population separately. The optimal temperature (T_opt_) for each thermal reaction norm was calculated from the formula T_opt_ = − b/2a, where *b* is the slope, and *a* the quadratic coefficient of the fitted quadratic function^77^. The mean values and confidence intervals for optimal temperatures, maximum values, performance breadths and coefficients of fitted quadratic functions were obtained by nonparametric bootstrapping: (1) we used random resampling with replacement, (2) fitted a quadratic function, (3) calculated the parameters, and (4) repeated this procedure 1000 times. To test whether the pairwise differences between the estimated parameters of the three acclimation groups are statistically significant (α = 0.05), we assessed bootstrap confidence intervals for each pairwise difference (if a given 95% confidence interval for differences between two groups includes zero, then the difference is not statistically significant at the 0.05 significance level)^78^. To examine the relationship between temperature and lipid droplet size and the relative cell area occupied by lipid droplets, we first fitted linear, quadratic, and cubic functions to our data (using mean values), and based on the Akaike information criterion (AICc), we chose the function with the lowest AICc value. In the case of lipid droplet size, the AICc value for a cubic function fitted to data obtained from the Slovak population was slightly lower than for a quadratic function, which has scored the lowest AICc value in the Indian population; however, for the sake of consistency and to facilitate comparisons, we used a quadratic function for both populations. The relationship between temperature and the relative cell area covered by lipid droplets was best described by a quadratic function in both populations. Next, we analysed lipid droplet size and the relative cell area occupied by lipid droplets by multiple nonlinear (quadratic) regression analyses with one categorical (‘population’), two continuous factors (‘test temperature’, ‘(test temperature)^2^’), and two interactions (‘population × test temperature’, ‘population × (test temperature)^2^’). Finally, we calculated Pearson's correlation coefficients (Pearson's r) between the fat content and the lipid droplet size, and the fat content and the relative cell area occupied by lipid droplets. All statistical analyses were performed using JMP v.15 (SAS, Raleigh, NC, USA) (multiple nonlinear regression analyses, curve fitting) and Excel (Microsoft) (nonparametric bootstrapping). Graphs were generated in GraphPad Prism 8 (GraphPad Software Inc.) and Excel (Microsoft).

## Supplementary information

Supplementary Information.

## Data Availability

The datasets generated during the current study are available from the corresponding author on reasonable request.
