# Supplementary material for: Acclimation temperature affects thermal reaction norms for energy reserves in Drosophila
Source: Sci Rep. 2020 Dec 10;10:21681. doi: 10.1038/s41598-020-78726-z (PMC7729904; doi:10.1038/s41598-020-78726-z)
Supplement: Supplementary file 1 — Supplementary Information. [file 41598_2020_78726_MOESM1_ESM.pdf]

Supplementary Information

Acclimation temperature affects thermal reaction norms for energy reserves in *Drosophila*

Peter Klepsatel, Thirnahalli Nagaraj Girish and Martina Gáliková

Supplementary Figures and Tables

Supplementary Figure S1. The effect of acclimation temperature on thermal reaction norms for fat stores. The fat content normalised to protein content (µg glycerides per mg protein) in flies from India (a) and Slovakia (b). Data points are mean values ± s.e.m.. Lines represent a quadratic fit to data with the 95% confidence band. For statistical analyses, see Supplementary Table S3-S5.

Supplementary Figure S2. The maximum values (i.e. values at the optimal temperature) for fat reserves estimated based on (a) the absolute fat content (µg glycerides per fly), and (b) the fat content normalised to protein content (µg glycerides per mg protein). Error bars represent 95% confidence intervals. Values with different letters are significantly different from each other ($\alpha = 0.05$).

Supplementary Figure S3. The effect of acclimation temperature on thermal reaction norms for the relative changes in the the fat content normalised to protein content (calculated as a ratio between the end values and the mean of initial values) in flies from India (a) and Slovakia (b). Data points are mean values ± s.e.m.. Lines represent a quadratic fit to data with the 95% confidence band. For statistical analyses, see Supplementary Table S3-S5.

Supplementary Figure S4. The optimal temperatures for fat reserves estimated based on (a) the fat content normalised to protein content (µg glycerides per mg protein), and (b) the relative changes in the fat content normalised to protein content. Error bars represent 95% confidence intervals. Values with different letters are significantly different from each other ($\alpha = 0.05$).

Supplementary Figure S5. The thermal performance breadth at 75% of maximum for fat reserves estimated based on (a) the absolute fat content (µg glycerides per fly), (b) the relative changes in the absolute fat content, (c) the fat content normalised to protein content (µg glycerides per mg protein), and (d) the relative changes in the fat content normalised to protein content. Error bars represent 95% confidence intervals. Values with different letters are significantly different from each other ($\alpha = 0.05$).

Supplementary Figure S6. The estimated coefficients of fitted quadratic functions ($y = ax^2 + bx + c$, where *a* is quadratic coefficient, *b* is slope, *c* is intercept, x is temperature, and y is the value of energy stores**) for fat reserves estimated based on (a, b, c) the absolute fat content (µg glycerides per fly), and (d, e, f) the relative changes in the absolute fat content.** (a, d) Quadratic coeficient, (b, e) slope, (c, f) intercept. Error bars represent 95% confidence intervals. Values with different letters are significantly different from each other ($\alpha = 0.05$).

Supplementary Figure S7. The estimated coefficients of fitted quadratic functions ($y = ax^2 + bx + c$, where *a* is quadratic coefficient, *b* is slope, *c* is intercept, x is temperature, and y is the value of energy stores**) for fat reserves estimated based on (a, b, c) the fat content**

normalised to protein content (µg glycerides per mg protein), and (d, e, f) the relative changes in the fat content normalised to protein content. (a, d) Quadratic coeficient, (b, e) slope, (c, f) intercept. Error bars represent 95% confidence intervals. Values with different letters are significantly different from each other (α = 0.05).

Supplementary Figure S8. The effect of acclimation temperature on thermal reaction norms for glycogen reserves. The glycogen content normalised to protein content (µg glycogen per mg protein) in flies from India (a) and Slovakia (b). Data points are mean values ± s.e.m.. Lines represent a quadratic fit to data with the 95% confidence band. For statistical analyses, see Supplementary Table S8-S10.

Supplementary Figure S9. The maximum values (i.e. values at the optimal temperature) for glycogen stores estimated based on (a) the absolute glycogen content (µg glycogen per fly), and (b) the glycogen content normalised to protein content (µg glycogen per mg protein). Error bars represent 95% confidence intervals. Values with different letters are significantly different from each other (α = 0.05).

Supplementary Figure S10. The effect of acclimation temperature on thermal reaction norms for the relative changes in the the glycogen content normalised to protein content (calculated as a ratio between the end values and the mean of initial values) in flies from India (a) and Slovakia (b). Data points are mean values ± s.e.m.. Lines represent a quadratic fit to data with the 95% confidence band. For statistical analyses, see Supplementary Table S8-S10.

Supplementary Figure S11. The optimal temperatures for glycogen stores estimated based on (a) the glycogen content normalised to protein content (µg glycogen per mg protein), and (b) the relative changes in the glycogen content normalised to protein content. Error bars represent 95% confidence intervals. Values with different letters are significantly different from each other (α = 0.05).

Supplementary Figure S12. The thermal performance breadth at 75% of maximum for glycogen stores estimated based on (a) the absolute glycogen content (µg glycogen per fly), (b) the relative changes in the absolute glycogen content, (c) the glycogen content normalised to protein content (µg glycogen per mg protein), and (d) the relative changes in the glycogen content normalised to protein content. Error bars represent 95% confidence intervals. Values with different letters are significantly different from each other (α = 0.05).

Supplementary Figure S13. The estimated coefficients of fitted quadratic functions ($y = ax^2 + bx + c$, where a is quadratic coefficient, b is slope, c is intercept, x is temperature, and y is the value of energy stores**) for glycogen stores estimated based on (a, b, c) the absolute glycogen content (µg glycogen per fly), and (d, e, f) the relative changes in the absolute glycogen content.** (a, d) Quadratic coeficient, (b, e) slope, (c, f) intercept. Error bars represent 95% confidence intervals. Values with different letters are significantly different from each other (α = 0.05).

Supplementary Figure S14. The estimated coefficients of fitted quadratic functions ($y = ax^2 + bx + c$, where a is quadratic coefficient, b is slope, c is intercept, x is temperature, and y is the value of energy stores**) for glycogen stores estimated based on (a, b, c) the glycogen content normalised to protein content (µg glycogen per mg protein), and (d, e, f) the**

relative changes in the glycogen content normalised to protein content. (a, d) Quadratic coeficient, (b, e) slope, (c, f) intercept. Error bars represent 95% confidence intervals. Values with different letters are significantly different from each other ($\alpha = 0.05$).

Supplementary Figure S1.

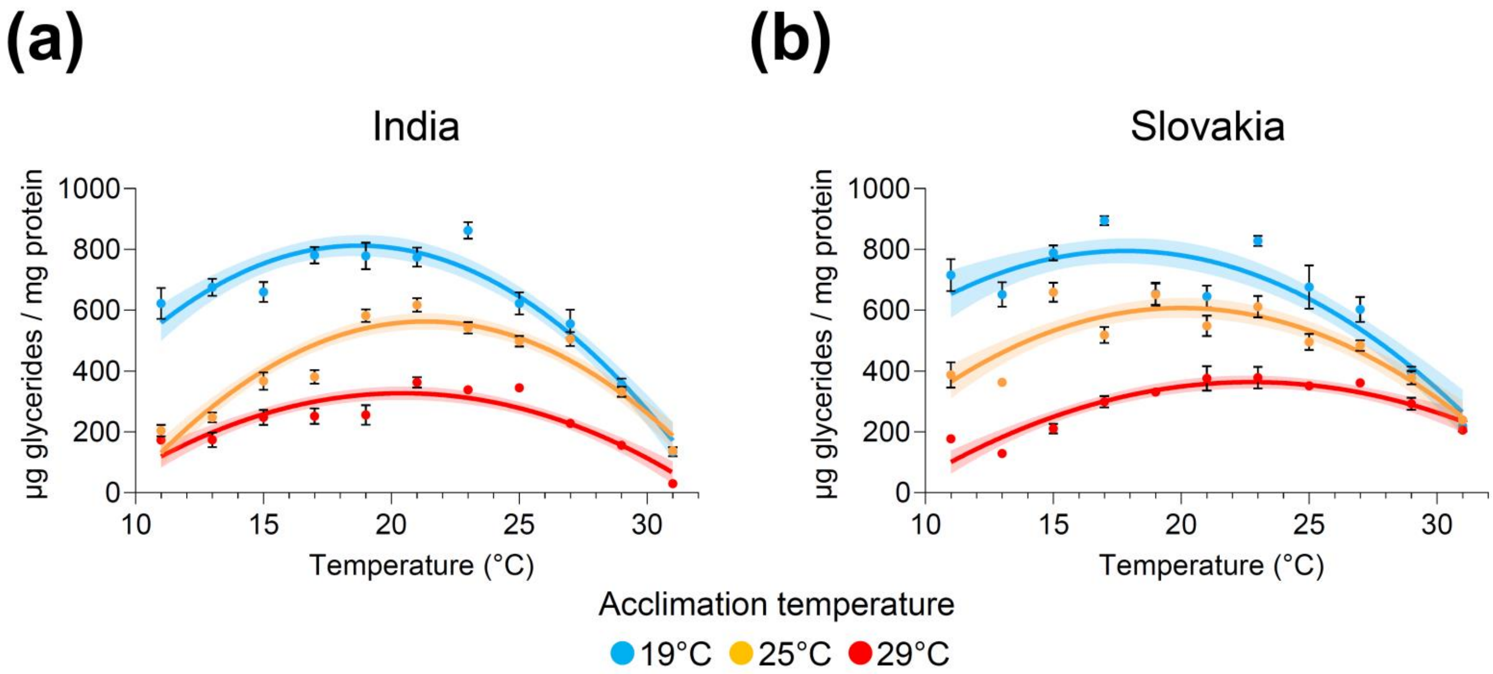

Supplementary Figure S2.

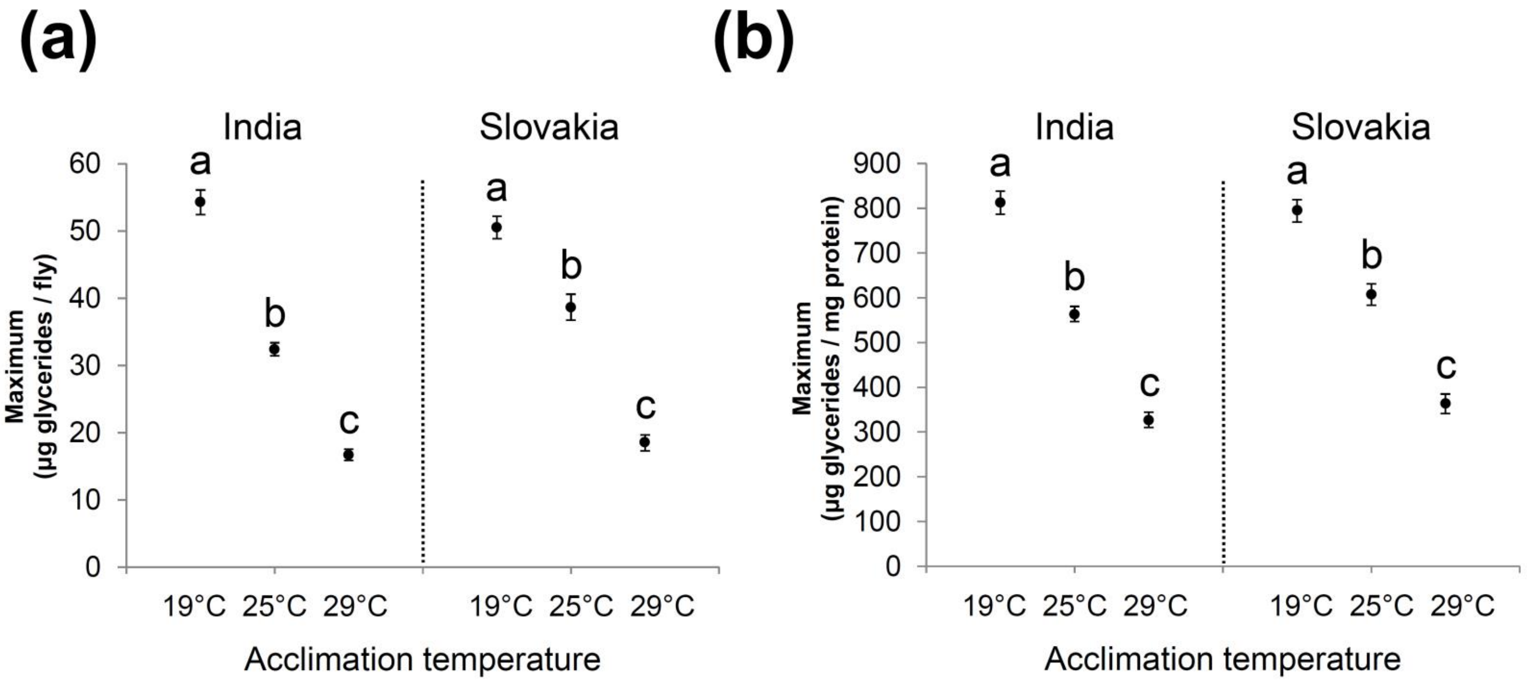

Supplementary Figure S3.

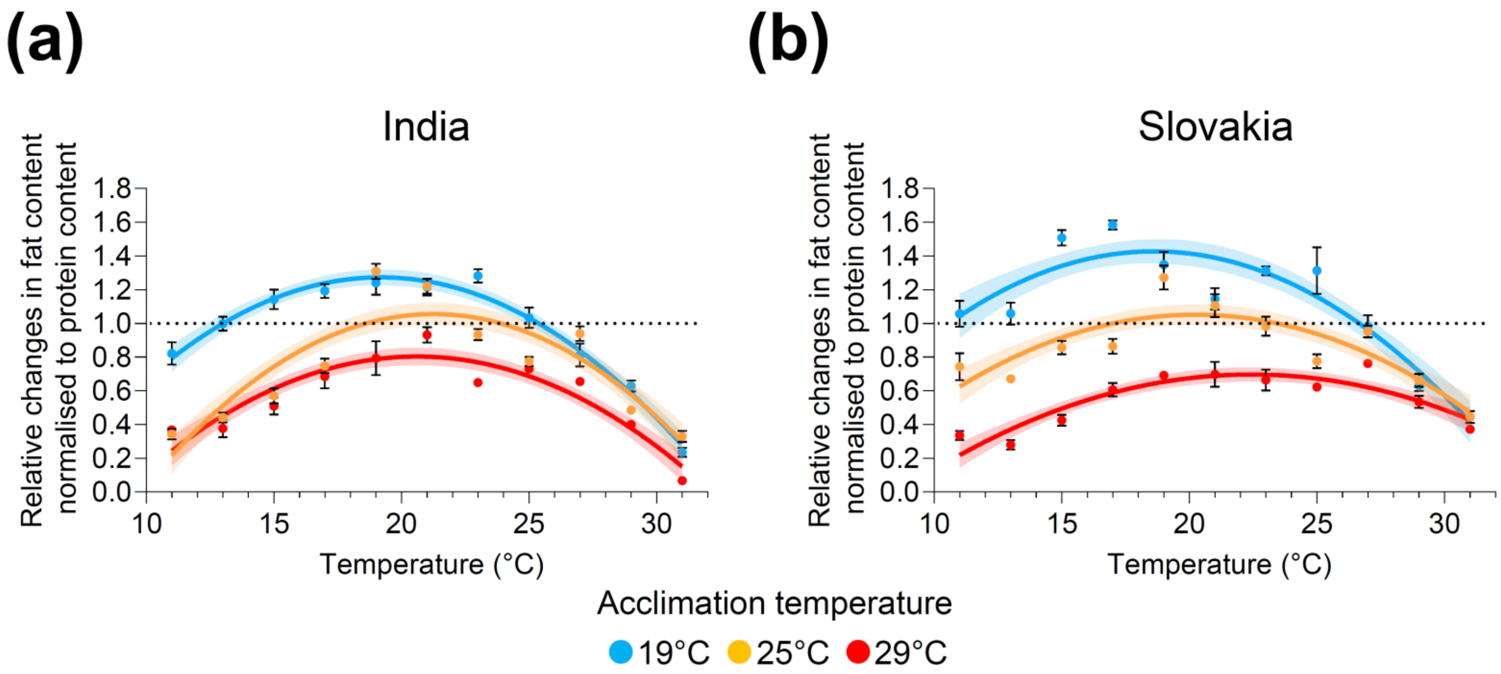

Supplementary Figure S4.

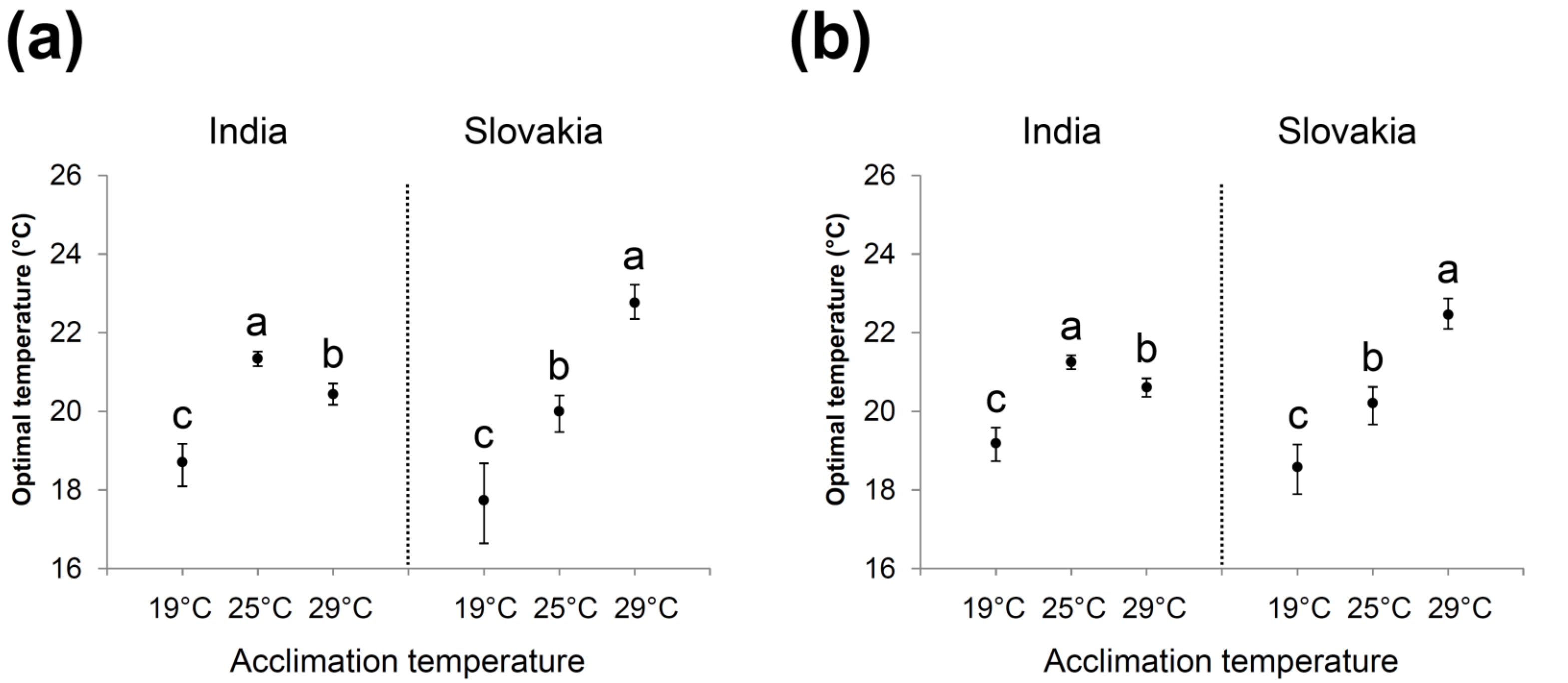

Supplementary Figure S5.

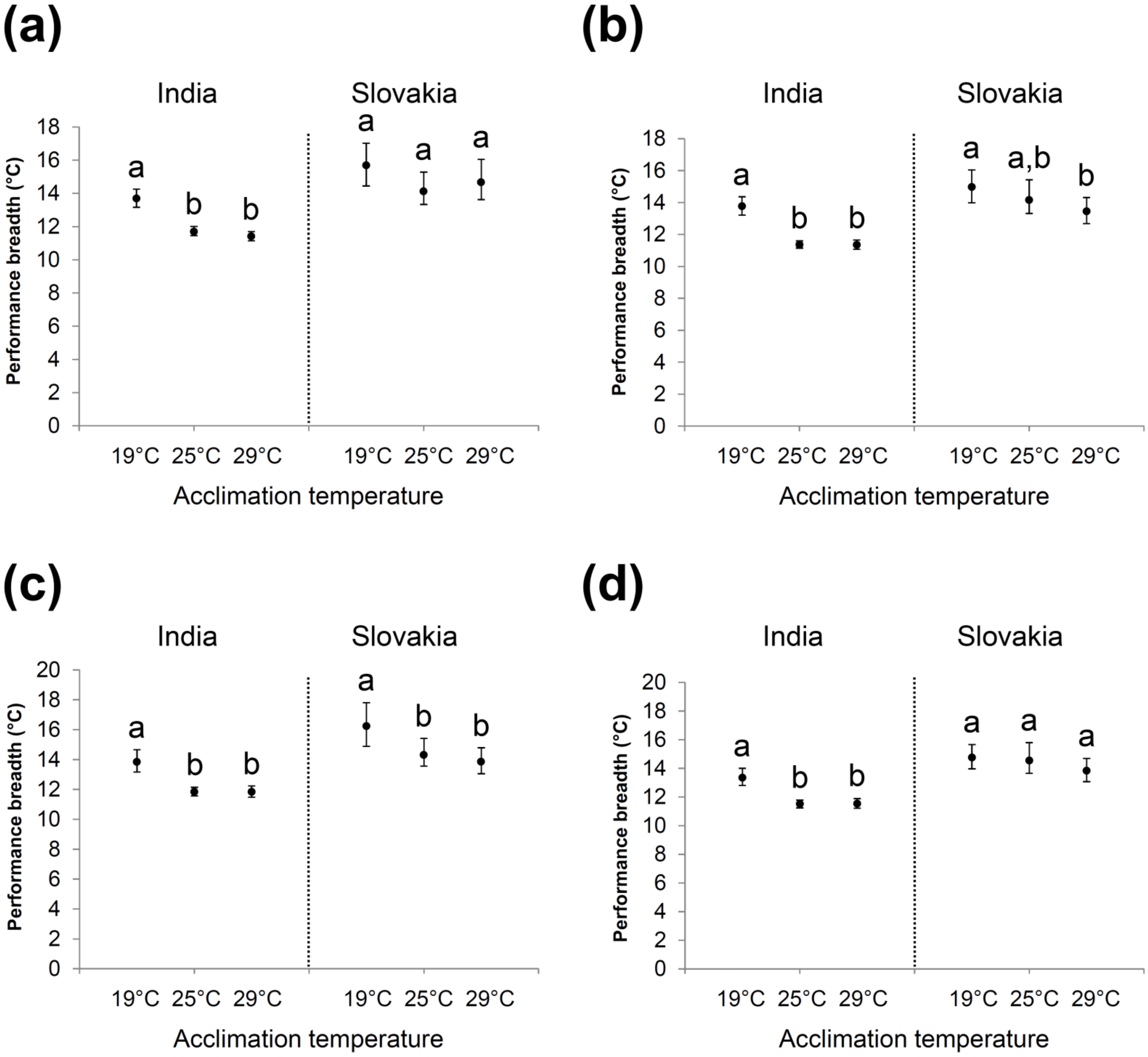

Supplementary Figure S6.

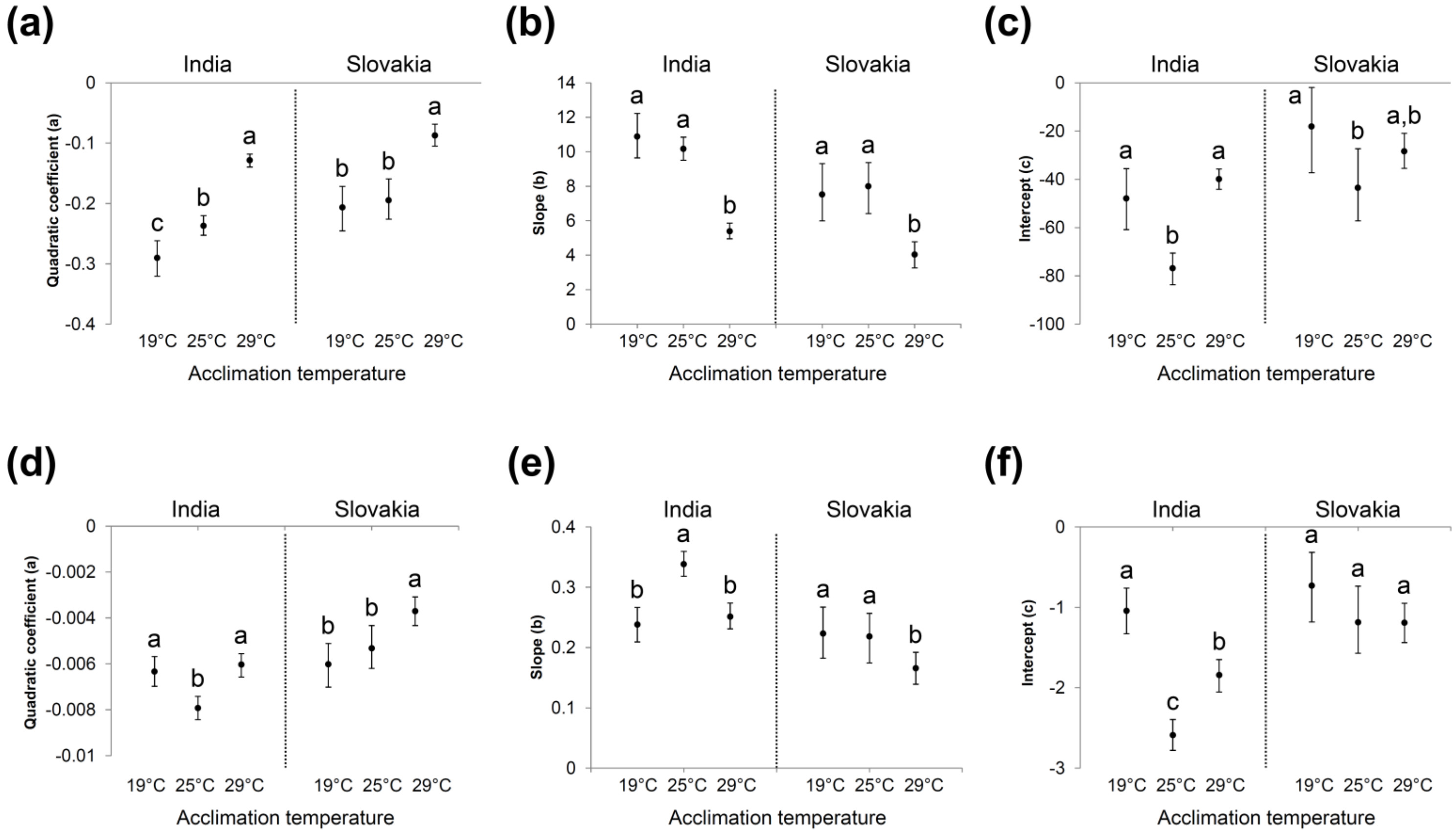

Supplementary Figure S7.

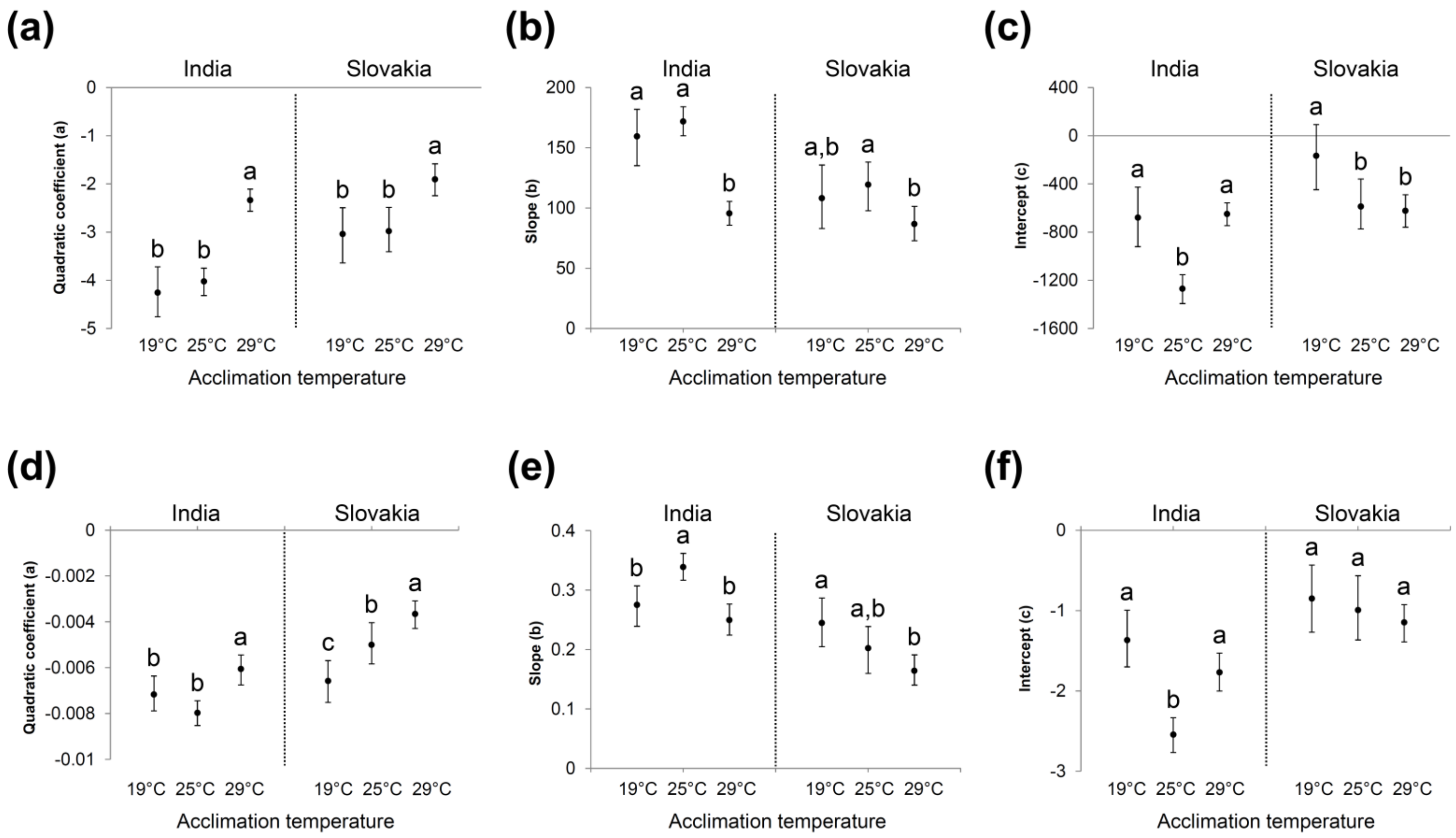

Supplementary Figure S8.

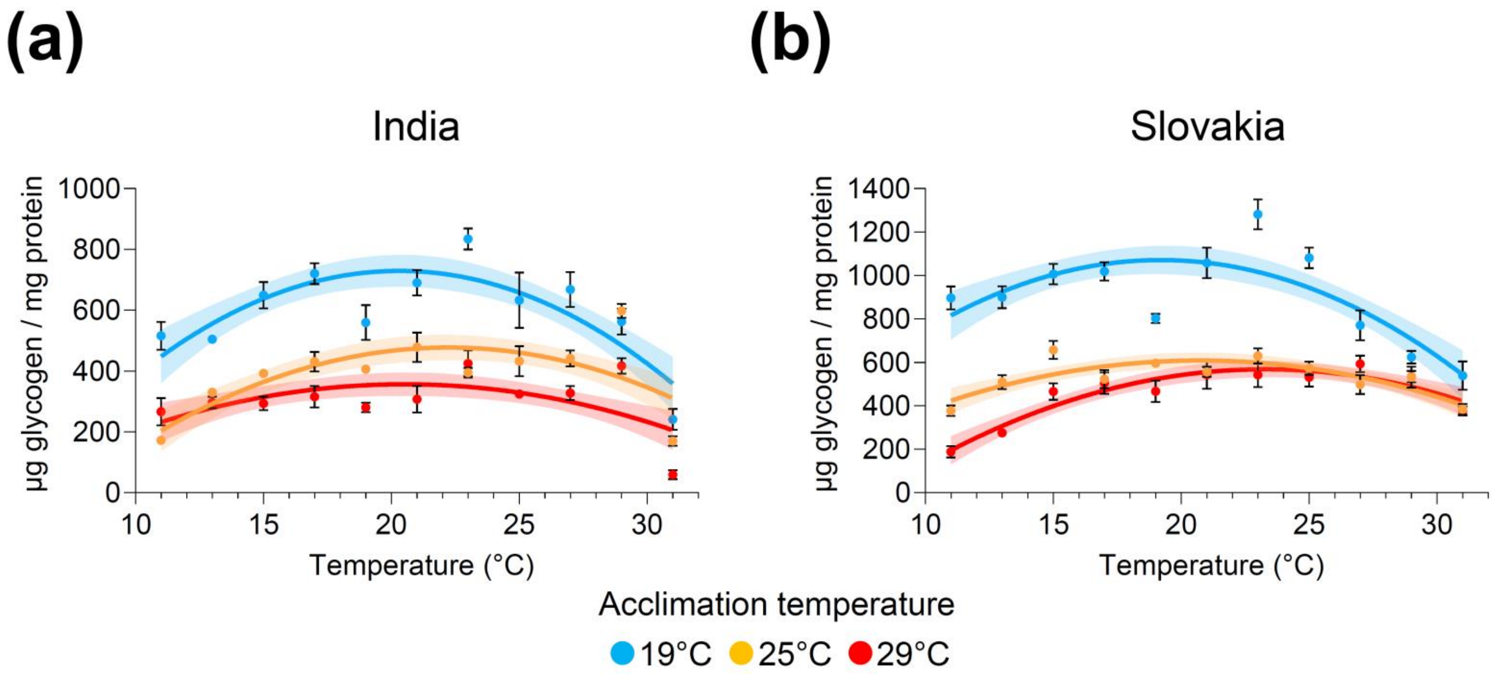

Supplementary Figure S9.

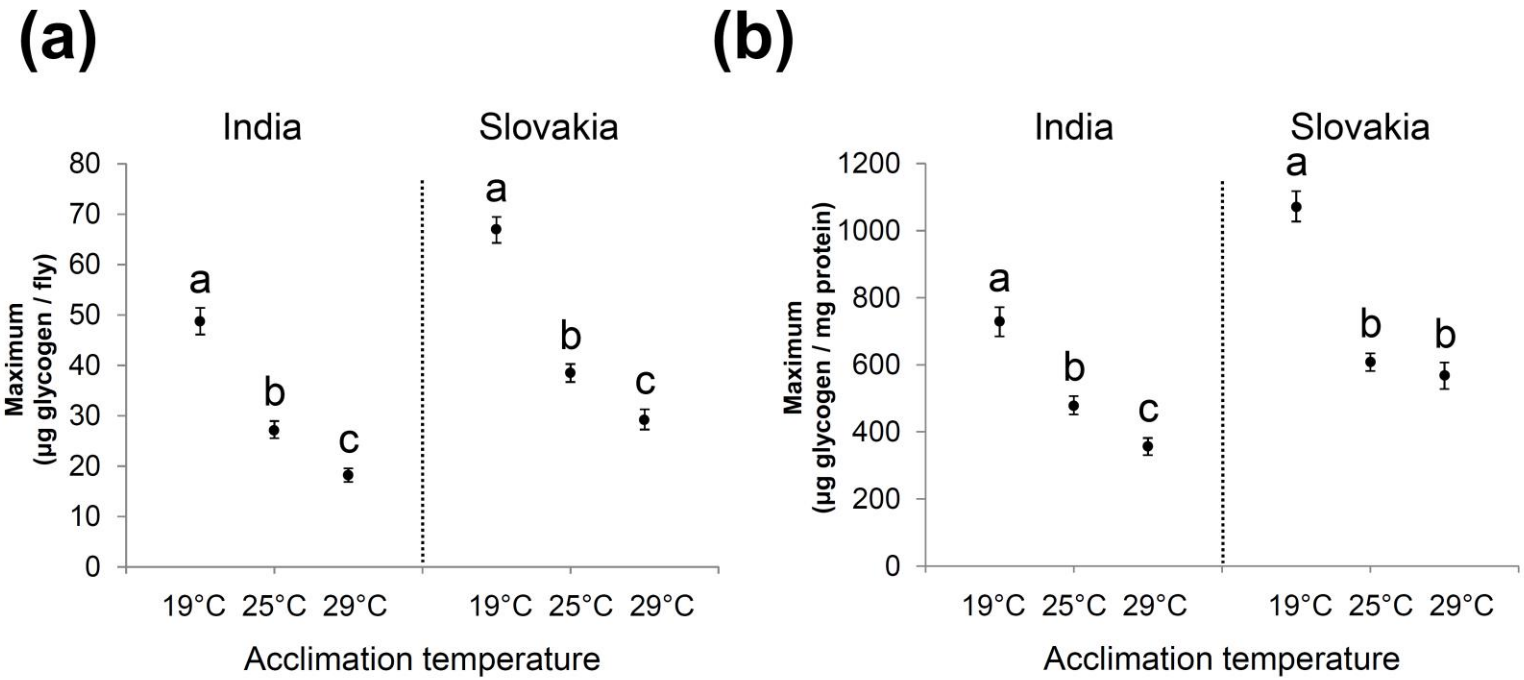

Supplementary Figure S10.

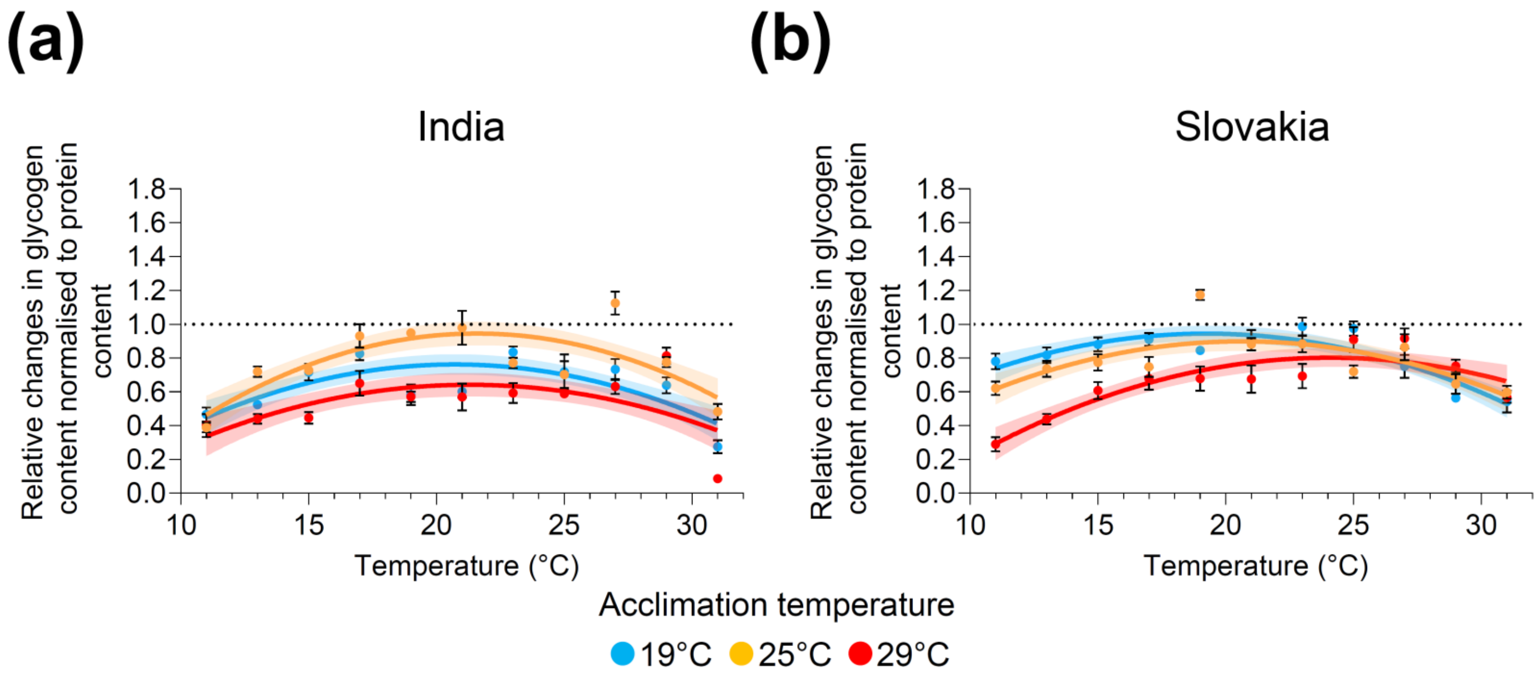

Supplementary Figure S11.

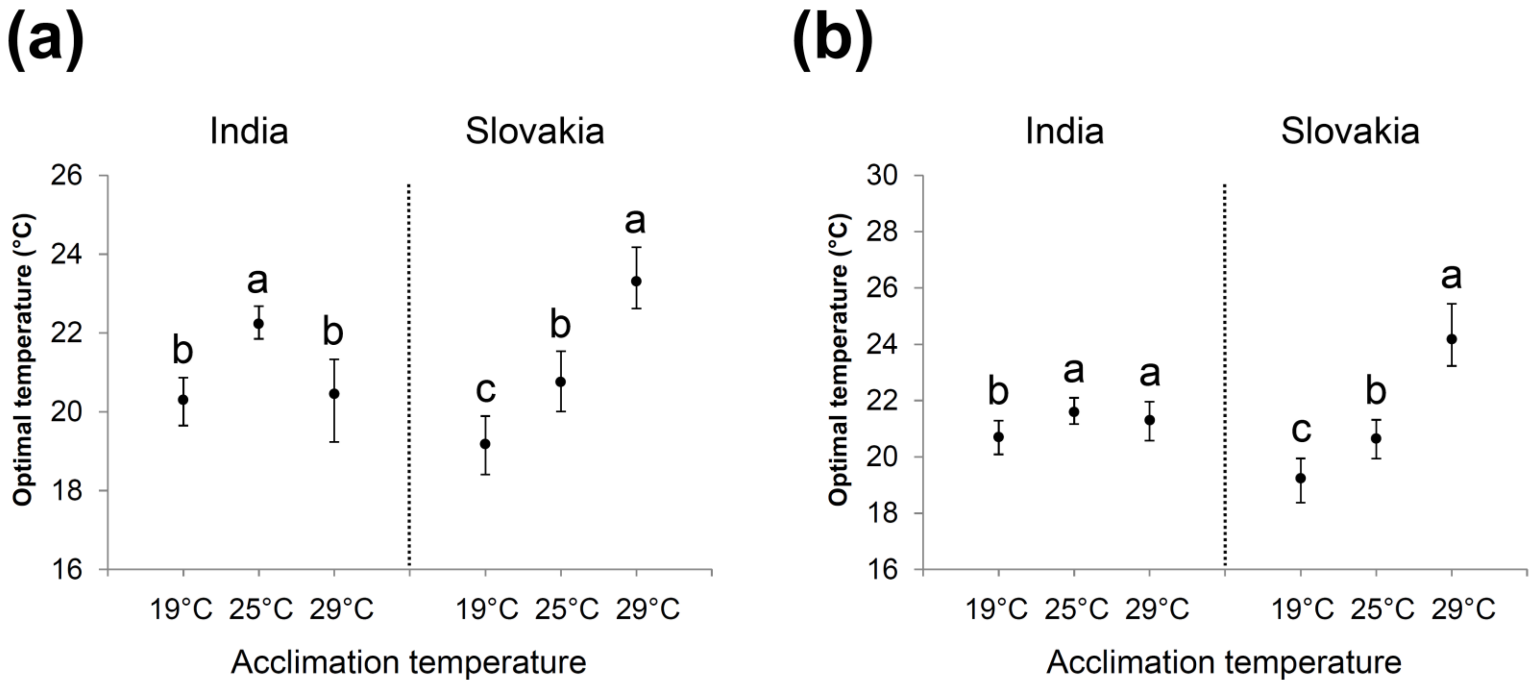

Supplementary Figure S12.

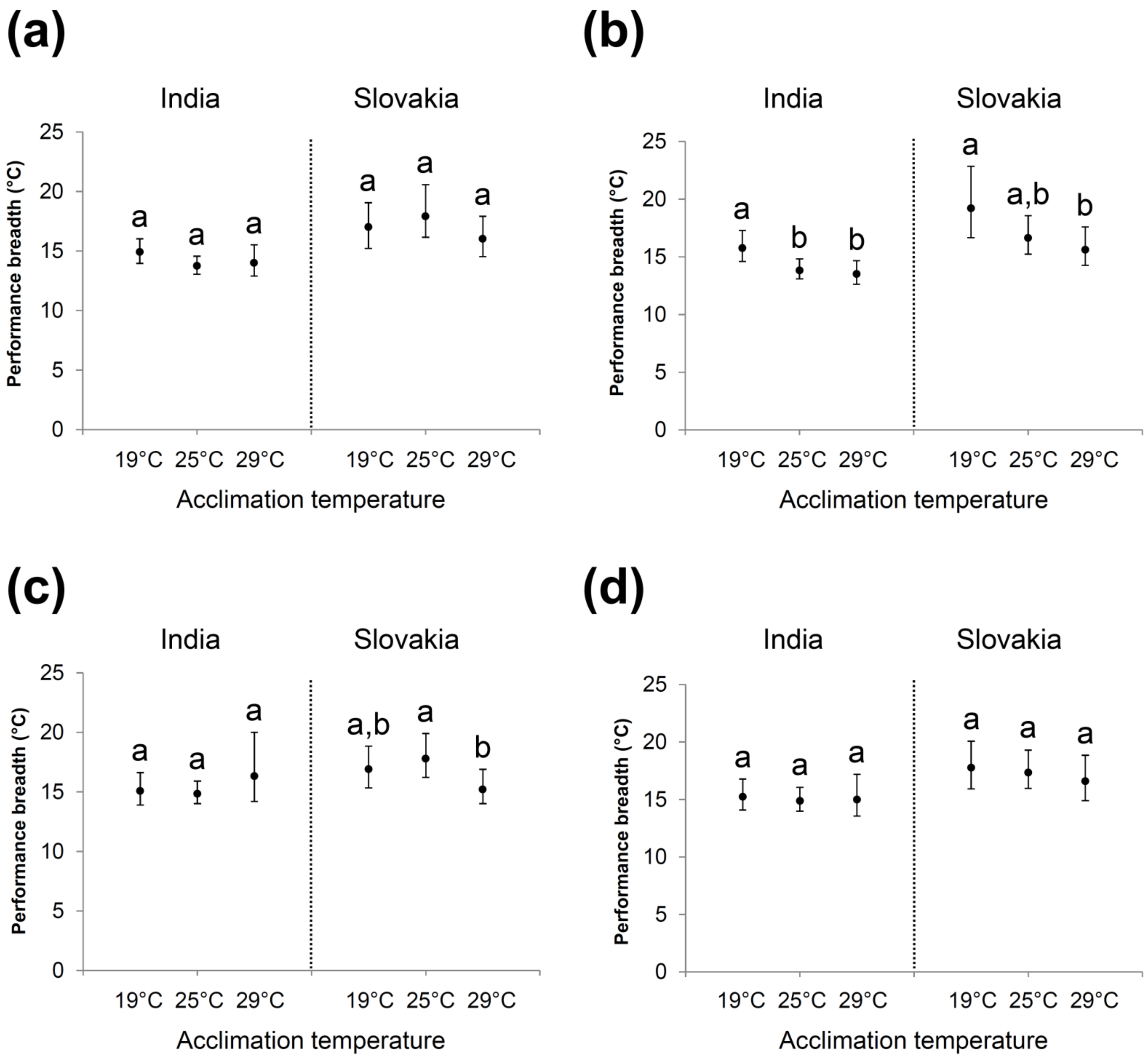

Supplementary Figure S13.

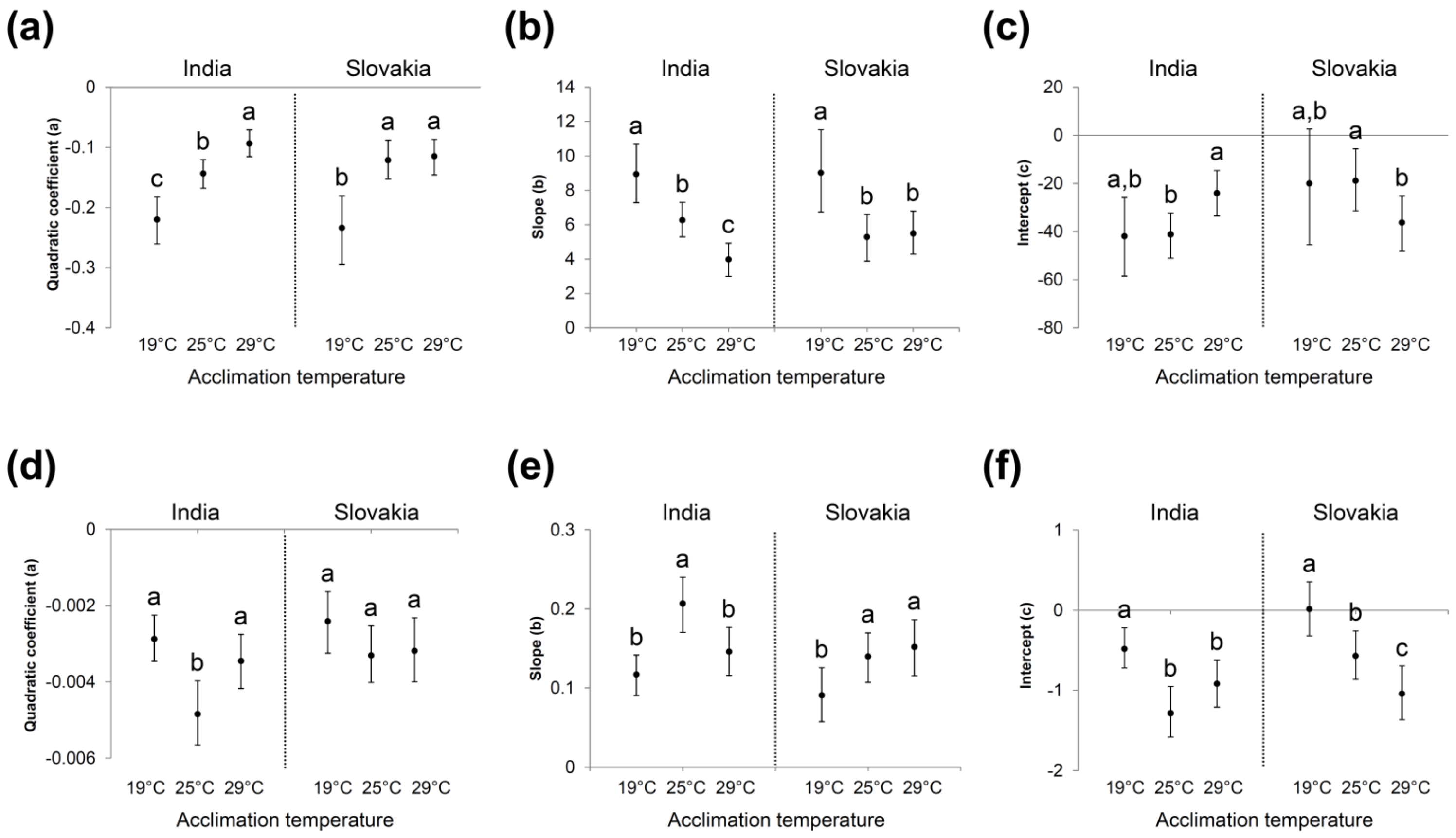

Supplementary Figure S14.

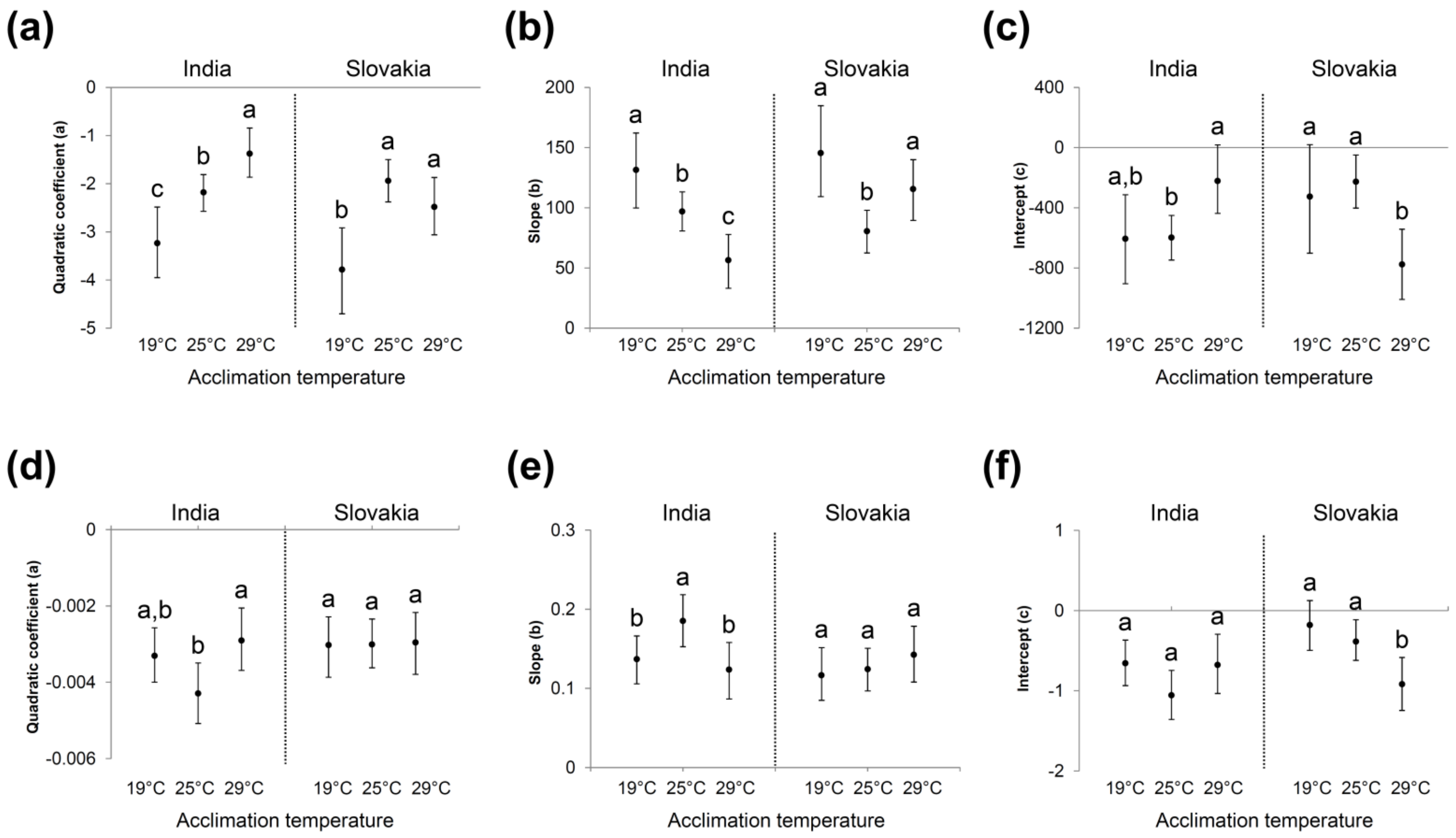

Supplementary Table S1. Multiple nonlinear (quadratic) regression analyses of the fat content (μg glycerides per fly). *df* - degrees of freedom; SSQ - the sum of squares for each source of variation.

**Source of variation**	***df***	**SSQ**	***F*-ratio**	***P*-value**
Population	1	1193.76	48.20	< 0.0001
Acclimation temperature	2	50768.03	1024.97	< 0.0001
Test temperature	1	15856.33	640.25	< 0.0001
(Test temperature)^2	1	17642.09	712.36	< 0.0001
Population × Acclimation temperature	2	1192.91	24.08	< 0.0001
Population × Test temperature	1	356.17	14.38	< 0.0001
Population × (Test temperature)^2	1	377.66	15.25	< 0.0001
Acclimation temperature × Test temperature	2	1162.66	23.47	< 0.0001
Acclimation temperature × (Test temperature)^2	2	1732.54	34.98	< 0.0001
Population × Acclimation temperature × Test temperature	2	48.77	0.98	0.37
Population × Acclimation temperature × (Test temperature)^2	2	49.99	1.01	0.37
Error	376	9311.91	-	-

Supplementary Table S2. Multiple nonlinear (quadratic) regression analyses of the relative changes in the fat content (the end values divided by the mean of initial values). *df* - degrees of freedom; SSQ - the sum of squares for each source of variation.

**Source of variation**	***df***	**SSQ**	***F*-ratio**	***P*-value**
Population	1	0.79	35.22	< 0.0001
Acclimation temperature	2	14.20	315.61	< 0.0001
Test temperature	1	15.46	687.32	< 0.0001
$(\text{Test temperature})^2$	1	16.88	750.19	< 0.0001
Population × Acclimation temperature	2	0.63	14.06	< 0.0001
Population × Test temperature	1	0.36	16.21	< 0.0001
Population × $(\text{Test temperature})^2$	1	0.37	16.59	< 0.0001
Acclimation temperature × Test temperature	2	0.23	5.17	0.006
Acclimation temperature × $(\text{Test temperature})^2$	2	0.27	5.99	0.003
Population × Acclimation temperature × Test temperature	2	0.13	2.88	0.058
Population × Acclimation temperature × $(\text{Test temperature})^2$	2	0.13	2.84	0.060
Error	376	8.46	-	-

Supplementary Table S3. Multiple nonlinear (quadratic) regression analyses of the fat content normalised to protein content (µg glycerides per mg protein). *df* - degrees of freedom; SSQ - the sum of squares for each source of variation.

**Source of variation**	***df***	**SSQ**	***F*-ratio**	***P*-value**
Population	1	258168.3	35.69	< 0.0001
Acclimation temperature	2	9116968.5	630.22	< 0.0001
Test temperature	1	4122303.6	569.92	< 0.0001
(Test temperature)^2	1	4652130.8	643.17	< 0.0001
Population × Acclimation temperature	2	63466.4	4.39	0.013
Population × Test temperature	1	94018.8	13.00	0.0004
Population × (Test temperature)^2	1	97047.6	13.42	0.0003
Acclimation temperature × Test temperature	2	149044.1	10.30	< 0.0001
Acclimation temperature × (Test temperature)^2	2	231130.9	15.98	< 0.0001
Population × Acclimation temperature × Test temperature	2	27342.9	1.89	0.15
Population × Acclimation temperature × (Test temperature)^2	2	13324.2	0.92	0.40
Error	376	2719672.7	-	-

Supplementary Table S4. Multiple nonlinear (quadratic) regression analyses of the relative changes in the fat content normalised to protein content (the end values divided by the mean of initial values). *df* - degrees of freedom; SSQ - the sum of squares for each source of variation.

**Source of variation**	***df***	**SSQ**	***F*-ratio**	***P*-value**
Population	1	0.71	28.63	< 0.0001
Acclimation temperature	2	15.86	318.33	< 0.0001
Test temperature	1	16.33	655.83	< 0.0001
$(\text{Test temperature})^2$	1	17.96	720.92	< 0.0001
Population × Acclimation temperature	2	0.56	11.20	< 0.0001
Population × Test temperature	1	0.47	18.92	< 0.0001
Population × $(\text{Test temperature})^2$	1	0.47	18.98	< 0.0001
Acclimation temperature × Test temperature	2	0.21	4.22	0.015
Acclimation temperature × $(\text{Test temperature})^2$	2	0.37	7.45	0.0007
Population × Acclimation temperature × Test temperature	2	0.13	2.65	0.072
Population × Acclimation temperature × $(\text{Test temperature})^2$	2	0.13	2.62	0.07
Error	376	9.36	-	-

Supplementary Table S5. Multiple nonlinear (quadratic) regression analyses of the fat content (µg glycerides per fly), the relative changes in the fat content, the fat content normalised to protein content (µg glycerides per mg protein), and the relative changes in the fat content normalised to protein content. *df* - degrees of freedom; SSQ - the sum of squares for each source of variation.

Population	Trait	Source of variation	*df*	SSQ	*F*-ratio	*P*-value
India	Fat content	Acclimation temperature	2	29661.15	762.75	< 0.0001
		Test temperature	1	10692.52	549.93	< 0.0001
		$(\text{Test temperature})^2$	1	11778.06	605.76	< 0.0001
		Acclimation temperature × Test temperature	2	822.77	21.16	< 0.0001
		Acclimation temperature × $(\text{Test temperature})^2$	2	1123.08	28.88	< 0.0001
		Error	189	3674.81	-	-
	Relative changes in fat content	Acclimation temperature	2	4.74	117.17	< 0.0001
		Test temperature	1	10.49	519.30	< 0.0001
		$(\text{Test temperature})^2$	1	11.31	559.90	< 0.0001
		Acclimation temperature × Test temperature	2	0.27	6.78	0.001
		Acclimation temperature × $(\text{Test temperature})^2$	2	0.17	4.26	0.016
		Error	189	3.82	-	-
	Fat content normalised to protein content	Acclimation temperature	2	4979814.1	414.92	< 0.0001
		Test temperature	1	2785374.0	464.15	< 0.0001
		$(\text{Test temperature})^2$	1	3095647.9	515.86	< 0.0001
		Acclimation temperature × Test temperature	2	154567.4	12.88	< 0.0001
		Acclimation temperature × $(\text{Test temperature})^2$	2	181277.9	15.10	< 0.0001
		Error	189	1134186.6	-	-
	Relative changes in the fat content normalised to protein content	Acclimation temperature	2	5.40	118.92	< 0.0001
		Test temperature	1	11.40	502.02	< 0.0001
		$(\text{Test temperature})^2$	1	12.32	542.64	< 0.0001
		Acclimation temperature × Test temperature	2	0.20	4.30	0.015
		Acclimation temperature × $(\text{Test temperature})^2$	2	0.15	3.37	0.037
		Error	189	4.29	-	-
Slovakia	Fat content	Acclimation temperature	2	22354.45	370.78	< 0.0001
		Test temperature	1	5619.53	186.42	< 0.0001
		$(\text{Test temperature})^2$	1	6328.20	209.93	< 0.0001
		Acclimation temperature × Test temperature	2	401.35	6.66	0.0016
		Acclimation temperature × $(\text{Test temperature})^2$	2	671.40	11.14	< 0.0001
		Error	187	5637.10	-	-
	Relative changes in fat content	Acclimation temperature	2	10.05	202.49	< 0.0001
		Test temperature	1	5.43	218.97	< 0.0001
		$(\text{Test temperature})^2$	1	6.02	242.64	< 0.0001
		Acclimation temperature × Test temperature	2	0.09	1.79	0.17
		Acclimation temperature × $(\text{Test temperature})^2$	2	0.22	4.47	0.013
		Error	187	4.64	-	-
	Fat content normalised to protein content	Acclimation temperature	2	4195347.7	247.41	< 0.0001
		Test temperature	1	1457015.2	171.85	< 0.0001
		$(\text{Test temperature})^2$	1	1676063.7	197.68	< 0.0001
		Acclimation temperature × Test temperature	2	24700.4	1.46	0.24
		Acclimation temperature × $(\text{Test temperature})^2$	2	65656.8	3.87	0.023
		Error	187	1585486.1	-	-
	Relative changes in the fat content normalised to protein content	Acclimation temperature	2	10.95	201.84	< 0.0001
		Test temperature	1	5.52	203.50	< 0.0001
		$(\text{Test temperature})^2$	1	6.20	228.64	< 0.0001
		Acclimation temperature × Test temperature	2	0.15	2.68	0.07
		Acclimation temperature × $(\text{Test temperature})^2$	2	0.34	6.32	0.0065
		Error	187	5.07	-	-

Supplementary Table S6. Multiple nonlinear (quadratic) regression analyses of the glycogen content (µg glycogen per fly). *df* - degrees of freedom; SSQ - the sum of squares for each source of variation.

**Source of variation**	***df***	**SSQ**	***F*-ratio**	***P*-value**
Population	1	16120.92	338.72	< 0.0001
Acclimation temperature	2	59815.16	628.40	< 0.0001
Test temperature	1	11503.28	241.70	< 0.0001
(Test temperature)^2	1	11693.51	245.70	< 0.0001
Population × Acclimation temperature	2	1055.44	11.09	< 0.0001
Population × Test temperature	1	3.02	0.06	0.80
Population × (Test temperature)^2	1	2.46	0.05	0.82
Acclimation temperature × Test temperature	2	886.20	9.31	0.0001
Acclimation temperature × (Test temperature)^2	2	1343.70	14.12	< 0.0001
Population × Acclimation temperature × Test temperature	2	64.23	0.67	0.51
Population × Acclimation temperature × (Test temperature)^2	2	39.67	0.42	0.66
Error	373	17752.40	-	-

Supplementary Table S7. Multiple nonlinear (quadratic) regression analyses of the relative changes in the glycogen content (the end values divided by the mean of initial values). *df* - degrees of freedom; SSQ - the sum of squares for each source of variation.

**Source of variation**	***df***	**SSQ**	***F*-ratio**	***P*-value**
Population	1	1.31	56.54	< 0.0001
Acclimation temperature	2	2.61	56.61	< 0.0001
Test temperature	1	5.48	237.51	< 0.0001
$(\text{Test temperature})^2$	1	5.46	236.50	< 0.0001
Population × Acclimation temperature	2	0.24	5.19	0.006
Population × Test temperature	1	0.06	2.44	0.12
Population × $(\text{Test temperature})^2$	1	0.07	2.96	0.09
Acclimation temperature × Test temperature	2	0.22	4.87	0.008
Acclimation temperature × $(\text{Test temperature})^2$	2	0.17	3.59	0.028
Population × Acclimation temperature × Test temperature	2	0.06	1.30	0.27
Population × Acclimation temperature × $(\text{Test temperature})^2$	2	0.04	0.83	0.44
Error	373	8.61	-	-

Supplementary Table S8. Multiple nonlinear (quadratic) regression analyses of the glycogen content normalised to protein content (µg glycogen per mg protein). *df* - degrees of freedom; SSQ - the sum of squares for each source of variation.

**Source of variation**	***df***	**SSQ**	***F*-ratio**	***P*-value**
Population	1	4000753	251.11	< 0.0001
Acclimation temperature	2	10152136	318.60	< 0.0001
Test temperature	1	2969256	186.36	< 0.0001
$(\text{Test temperature})^2$	1	3055424	191.77	< 0.0001
Population × Acclimation temperature	2	558162	17.52	< 0.0001
Population × Test temperature	1	24658	1.55	0.21
Population × $(\text{Test temperature})^2$	1	27676	1.74	0.19
Acclimation temperature × Test temperature	2	162495	5.10	0.0065
Acclimation temperature × $(\text{Test temperature})^2$	2	257162	8.07	0.0004
Population × Acclimation temperature × Test temperature	2	63612	2.00	0.14
Population × Acclimation temperature × $(\text{Test temperature})^2$	2	35591	1.12	0.33
Error	373	5942829	-	-

Supplementary Table S9. Multiple nonlinear (quadratic) regression analyses of the relative changes in the glycogen content normalised to protein content (the end values divided by the mean of initial values). *df* - degrees of freedom; SSQ - the sum of squares for each source of variation.

**Source of variation**	***df***	**SSQ**	***F*-ratio**	***P*-value**
Population	1	1.11	42.61	< 0.0001
Acclimation temperature	2	2.32	44.38	< 0.0001
Test temperature	1	5.20	198.78	< 0.0001
(Test temperature)^2	1	5.15	196.91	< 0.0001
Population × Acclimation temperature	2	0.54	10.32	< 0.0001
Population × Test temperature	1	0.03	1.11	0.29
Population × (Test temperature)^2	1	0.03	1.16	0.28
Acclimation temperature × Test temperature	2	0.04	0.76	0.47
Acclimation temperature × (Test temperature)^2	2	0.04	0.86	0.42
Population × Acclimation temperature × Test temperature	2	0.07	1.42	0.24
Population × Acclimation temperature × (Test temperature)^2	2	0.04	0.76	0.47
Error	373	9.75	-	-

Supplementary Table S10. Multiple nonlinear (quadratic) regression analyses of the glycogen content (µg glycogen per fly), the relative changes in the glycogen content, the glycogen content normalised to protein content (µg glycogen per mg protein), and the relative changes in the glycogen content normalised to protein content. *df* - degrees of freedom; SSQ - the sum of squares for each source of variation.

Population	Trait	Source of variation	*df*	SSQ	*F*-ratio	*P*-value
India	Glycogen content	Acclimation temperature	2	22558.61	268.99	< 0.0001
		Test temperature	1	5565.93	132.74	< 0.0001
		(Test temperature)^2	1	5677.98	135.41	< 0.0001
		Acclimation temperature × Test temperature	2	551.68	6.58	0.0017
		Acclimation temperature × (Test temperature)^2	2	654.13	7.80	0.0006
		Error	186	7799.22	-	-
	Relative changes in glycogen content	Acclimation temperature	2	1.81	33.66	< 0.0001
		Test temperature	1	3.33	123.86	< 0.0001
		(Test temperature)^2	1	3.38	125.71	< 0.0001
		Acclimation temperature × Test temperature	2	0.19	3.54	0.031
		Acclimation temperature × (Test temperature)^2	2	0.17	3.09	0.047
		Error	186	4.99	-	-
	Glycogen content normalised to protein content	Acclimation temperature	2	3058418.7	106.94	< 0.0001
		Test temperature	1	1226156.5	85.75	< 0.0001
		(Test temperature)^2	1	1250693.4	87.47	< 0.0001
		Acclimation temperature × Test temperature	2	130610.1	4.57	0.012
		Acclimation temperature × (Test temperature)^2	2	145123.7	5.07	0.007
		Error	186	2659682.0	-	-
	Relative changes in the glycogen content normalised to protein content	Acclimation temperature	2	1.94	30.91	< 0.0001
		Test temperature	1	3.00	95.72	< 0.0001
		(Test temperature)^2	1	2.98	95.19	< 0.0001
		Acclimation temperature × Test temperature	2	0.10	1.54	0.22
		Acclimation temperature × (Test temperature)^2	2	0.08	1.35	0.26
		Error	186	5.83	-	-
Slovakia	Glycogen content	Acclimation temperature	2	38275.61	359.56	< 0.0001
		Test temperature	1	5940.43	111.61	< 0.0001
		(Test temperature)^2	1	6018.01	113.07	< 0.0001
		Acclimation temperature × Test temperature	2	398.79	3.75	0.025
		Acclimation temperature × (Test temperature)^2	2	729.26	6.85	0.0013
		Error	187	9953.17	-	-
	Relative changes in glycogen content	Acclimation temperature	2	1.04	26.74	< 0.0001
		Test temperature	1	2.22	114.45	< 0.0001
		(Test temperature)^2	1	2.15	111.30	< 0.0001
		Acclimation temperature × Test temperature	2	0.09	2.45	0.089
		Acclimation temperature × (Test temperature)^2	2	0.04	0.98	0.38
		Error	187	3.62	-	-
	Glycogen content normalised to protein content	Acclimation temperature	2	7646660.7	217.77	< 0.0001
		Test temperature	1	1767854.0	100.69	< 0.0001
		(Test temperature)^2	1	1832435.8	104.37	< 0.0001
		Acclimation temperature × Test temperature	2	95523.1	2.72	0.069
		Acclimation temperature × (Test temperature)^2	2	147636.8	4.20	0.016
		Error	187	3283147.1	-	-
	Relative changes in the glycogen content normalised to protein content	Acclimation temperature	2	0.91	21.67	< 0.0001
		Test temperature	1	2.23	106.12	< 0.0001
		(Test temperature)^2	1	2.19	104.65	< 0.0001
		Acclimation temperature × Test temperature	2	0.02	0.41	0.67
		Acclimation temperature × (Test temperature)^2	2	0.0002	0.004	0.99
		Error	187	3.92	-	-

Supplementary Table S11. The Akaike information criterion (AICc) for different functions fitted to data on the temperature dependence of lipid droplet size and the relative cell area occupied by lipid droplets.

Trait	Population	Linear	Quadratic	Cubic
Lipid droplet size	India	38.07	**34.76**	34.84
	Slovakia	49.08	39.98	**38.38**
Rrelative cell area occupied by lipid droplets	India	-38.44	**-41.88**	-38.29
	Slovakia	-39.20	**-48.69**	-42.84

Supplementary Table S12. Multiple nonlinear (quadratic) regression analyses of the changes in lipid droplet size and the relative cell area occupied by lipid droplets (lipid droplet area/cell area). See Materials and methods for further details. *df* - degrees of freedom; SSQ - the sum of squares for each source of variation.

**Trait**	**Source of variation**	***df***	**SSQ**	***F*-ratio**	***P*-value**
Lipid droplet size	Population	1	792.22	170.91	< 0.0001
	Test temperature	1	1279.85	276.11	< 0.0001
	(Test temperature)^2	1	1195.33	257.88	< 0.0001
	Population × Test temperature	1	138.04	29.78	< 0.0001
	Population × (Test temperature)^2	1	118.85	25.64	< 0.0001
	Error	373	5988.73	-	-
Relative cell area occupied by lipid droplets	Population	1	0.20	31.95	< 0.0001
	Test temperature	1	0.53	86.64	< 0.0001
	(Test temperature)^2	1	0.61	99.62	< 0.0001
	Population × Test temperature	1	0.004	0.65	0.42
	Population × (Test temperature)^2	1	0.002	0.39	0.53
	Error	1292	7.96	-	-

Supplementary Table S13. Pairwaise (Pearson) correlations between the fat content (µg glycerides per fly) and the lipid droplet size, and the relative cell area occupied by lipid droplets.

		Lipid droplet size		Relative cell area occupied by lipid droplets	
		Pearson's r	*P*-value	Pearson's r	*P*-value
Fat content	India	0.8067	0.0027	0.6640	0.026
	Slovakia	0.5425	0.085	0.8264	0.0017
	Combined	0.6943	0.0003	0.7787	< 0.0001
